# Supplementary material for: Antiviral Activity of Anthranilamide Peptidomimetics against Herpes Simplex Virus 1 and a Coronavirus
Source: Antibiotics (Basel). 2023 Sep 12;12(9):1436. doi: 10.3390/antibiotics12091436 (PMC10525570; doi:10.3390/antibiotics12091436)

The Supplementary Data for-

Antiviral Activity of Anthranilamide Peptidomimetics against herpes simplex virus 1 and a coronavirus

Umme Laila Urmi[1*], Samuel Attard[2], Ajay Kumar Vijay[1], Mark D. P. Willcox[1*], Naresh Kumar[2], Salequl Islam[1,3], Rajesh Kuppusamy[1,2]

[1]School of Optometry and Vision Science, University of New South Wales, Sydney, NSW 2052, Australia
[2]School of Chemistry, University of New South Wales, Sydney, NSW 2052, Australia
[3]Department of Microbiology, Jahangirnagar University, Savar, Dhaka-1342, Bangladesh

---

Contents

^{1}H NMR spectra of (*S*)-*N*-(2-((1-((2-aminoethyl)amino)-3-(1*H*-indol-3-yl)-1-oxopropan-2-yl)carbamoyl)phenyl)-2-naphthamide (**1**) 3

^{13}C NMR spectra of (*S*)-*N*-(2-((1-((2-aminoethyl)amino)-3-(1*H*-indol-3-yl)-1-oxopropan-2-yl)carbamoyl)phenyl)-2-naphthamide (**1**).......... 4

^{1}H NMR spectra of (*S*)-*N*-(2-((1-((2-aminoethyl)amino)-3-(*1H*-indol-3-yl)-1-oxopropan-2-yl)carbamoyl)-4-fluorophenyl)-2-naphthamide (**2**) 5

^{13}C NMR spectra of (*S*)-*N*-(2-((1-((2-aminoethyl)amino)-3-(*1H*-indol-3-yl)-1-oxopropan-2-yl)carbamoyl)-4-fluorophenyl)-2-naphthamide (**2**) 7

^{1}H NMR spectra of (*S*)-*N*-(2-((1-((2-aminoethyl)amino)-3-(*1H*-indol-3-yl)-1-oxopropan-2-yl)carbamoyl)-4-chlorophenyl)-2-naphthamide (**3**).......... 8

^{13}C NMR spectra of (*S*)-*N*-(2-((1-((2-aminoethyl)amino)-3-(*1H*-indol-3-yl)-1-oxopropan-2-yl)carbamoyl)-4-chlorophenyl)-2-naphthamide (**3**) 9

^{1}H NMR spectra of (*S*)-*N*-(2-((1-((2-aminoethyl)amino)-3-(*1H*-indol-3-yl)-1-oxopropan-2-yl)carbamoyl)-4-bromophenyl)-2-naphthamide (**4**) 10

^{13}C NMR spectra of (*S*)-*N*-(2-((1-((2-aminoethyl)amino)-3-(1*H*-indol-3-yl)-1-oxopropan-2-yl)carbamoyl)-4-bromophenyl)-2-naphthamide (**4**) 11

^{1}H NMR spectra of (*S*)-*N*-(2-((1-((2-aminoethyl)amino)-3-(*1H*-indol-3-yl)-1-oxopropan-2-yl)carbamoyl)-4-methoxyphenyl)-2-naphthamide (**5**) 12

^{13}C NMR spectra of (*S*)-*N*-(2-((1-((2-aminoethyl)amino)-3-(*1H*-indol-3-yl)-1-oxopropan-2-yl)carbamoyl)-4-methoxyphenyl)-2-naphthamide (**5**) 13

^{1}H NMR spectra of (*S*)-*N*-(2-((1-((2-guanidinoethyl)amino)-3-(*1H*-indol-3-yl)-1-oxopropan-2-yl)carbamoyl)phenyl)-2-naphthamide (**6**).......... 14

^{13}C NMR spectra of (*S*)-*N*-(2-((1-((2-guanidinoethyl)amino)-3-(*1H*-indol-3-yl)-1-oxopropan-2-yl)carbamoyl)phenyl)-2-naphthamide (**6**).......... 15

^{1}H NMR spectra of (*S*)-*N*-(4-fluoro-2-((1-((2-guanidinoethyl)amino)-3-(*1H*-indol-3-yl)-1-oxopropan-2-yl)carbamoyl)phenyl)-2-naphthamide (**7**) 16

^{13}C NMR spectra of (*S*)-*N*-(4-fluoro-2-((1-((2-guanidinoethyl)amino)-3-(*1H*-indol-3-yl)-1-oxopropan-2-yl)carbamoyl)phenyl)-2-naphthamide (**7**) 17

^{1}H NMR spectra of (*S*)-*N*-(4-chloro-2-((1-((2-guanidinoethyl)amino)-3-(*1H*-indol-3-yl)-1-oxopropan-2-yl)carbamoyl)phenyl)-2-naphthamide (**8**) 18

^{13}C NMR spectra of (*S*)-*N*-(4-chloro-2-((1-((2-guanidinoethyl)amino)-3-(*1H*-indol-3-yl)-1-oxopropan-2-yl)carbamoyl)phenyl)-2-naphthamide (**8**) 19

^{1}H NMR spectra of (*S*)-*N*-(4-bromo-2-((1-((2-guanidinoethyl)amino)-3-(1*H*-indol-3-yl)-1-oxopropan-2-yl)carbamoyl)phenyl)-2-naphthamide (**9**) 20

^{13}C NMR spectra of (*S*)-*N*-(4-bromo-2-((1-((2-guanidinoethyl)amino)-3-(1*H*-indol-3-yl)-1-oxopropan-2-yl)carbamoyl)phenyl)-2-naphthamide (**9**) 21

^{1}H NMR spectra of (*S*)-*N*-(2-((1-((2-guanidinoethyl)amino)-3-(*1H*-indol-3-yl)-1-oxopropan-2-yl)carbamoyl)-4-methoxyphenyl)-2-naphthamide (**10**) 22

^{13}C NMR spectra of (*S*)-*N*-(2-((1-((2-guanidinoethyl)amino)-3-(*1H*-indol-3-yl)-1-oxopropan-2-yl)carbamoyl)-4-methoxyphenyl)-2-naphthamide (**10**) 23

^{1}H NMR of (*S*)-2-amino-*N*-(1-((2-aminoethyl)amino)-3-(1*H*-indol-3-yl)-1-oxopropan-2-yl)-5-(naphthalen-2-yl)benzamide (**11**) 24

^{13}C NMR of (*S*)-2-amino-*N*-(1-((2-aminoethyl)amino)-3-(1*H*-indol-3-yl)-1-oxopropan-2-yl)-5-(naphthalen-2-yl)benzamide (**11**)... 24

^{1}H NMR of (S)-4-amino-N-(1-((2-aminoethyl)amino)-3-(1H-indol-3-yl)-1-oxopropan-2-yl)-[1,1':4',1''-terphenyl]-3-carboxamide (**12**) 25

^{13}C NMR of (S)-4-amino-N-(1-((2-aminoethyl)amino)-3-(1H-indol-3-yl)-1-oxopropan-2-yl)-[1,1':4',1''-terphenyl]-3-carboxamide (**12**) 27

^{1}H NMR spectra of (S)-2-amino-N-(1-((2-aminoethyl)amino)-3-(1H-indol-3-yl)-1-oxopropan-2-yl)-5-(2,4-dimethoxypyrimidin-5-yl)benzamide (**13**)……………………..26

^{13}C NMR spectra of (S)-2-amino-N-(1-((2-aminoethyl)amino)-3-(1H-indol-3-yl)-1-oxopropan-2-yl)-5-(2,4-dimethoxypyrimidin-5-yl)benzamide (**13**)…………………27

^{1}H NMR spectra of (S)-4-amino-N-(1-((2-aminoethyl)amino)-3-(1H-indol-3-yl)-1-oxopropan-2-yl)-[1,1':2',1''-terphenyl]-3-carboxamide (**14**)…………………………….28

^{13}C NMR spectra of(S)-4-amino-N-(1-((2-aminoethyl)amino)-3-(1H-indol-3-yl)-1-oxopropan-2-yl)-[1,1':2',1''-terphenyl]-3-carboxamide (**14**)……………………………….29

^{1}H NMR of *N*-(4-bromo-2-(((*S*)-1-((2-((*S*)-2,6-diaminohexanamido)ethyl)amino)-3-(1*H*-indol-3-yl)-1-oxopropan-2-yl)carbamoyl)phenyl)-2-naphthamide (**15**……….30

^{13}C NMR of *N*-(4-bromo-2-(((S)-1-((2-((S)-2,6-diaminohexanamido)ethyl)amino)-3-(1*H*-indol-3-yl)-1-oxopropan-2-yl)carbamoyl)phenyl)-2-naphthamide (**15**)……….31

^{1}HNMR of *N*-(4-bromo-2-(((S)-1-((2-((S)-2,6-diaminohexanamido)ethyl)amino)-3-(1*H*-indol-3-yl)-1-oxopropan-2-yl)carbamoyl)phenyl)-[1,1'-biphenyl]-3-carboxamide (**16**)..32

^{13}C NMR of *N*-(4-bromo-2-(((S)-1-((2-((S)-2,6-diaminohexanamido)ethyl)amino)-3-(1*H*-indol-3-yl)-1-oxopropan-2-yl)carbamoyl)phenyl)-[1,1'-biphenyl]-3-carboxamide (**16**).33

^{1}H NMR of *N*-(4-bromo-2-(((S)-1-((2-((S)-2,6-diaminohexanamido)ethyl)amino)-3-(1*H*-indol-3-yl)-1-oxopropan-2-yl)carbamoyl)phenyl)-[1,1'-biphenyl]-4-carboxamide (**17**).34

^{13}C NMR of *N*-(4-bromo-2-(((S)-1-((2-((S)-2,6-diaminohexanamido)ethyl)amino)-3-(1*H*-indol-3-yl)-1-oxopropan-2-yl)carbamoyl)phenyl)-[1,1'-biphenyl]-4-carboxamide (**17**).35

^{1}H NMR spectra of (*S*)-*N*-(2-((1-((2-aminoethyl)amino)-3-(1*H*-indol-3-yl)-1-oxopropan-2-yl)carbamoyl)phenyl)-2-naphthamide (**1**)

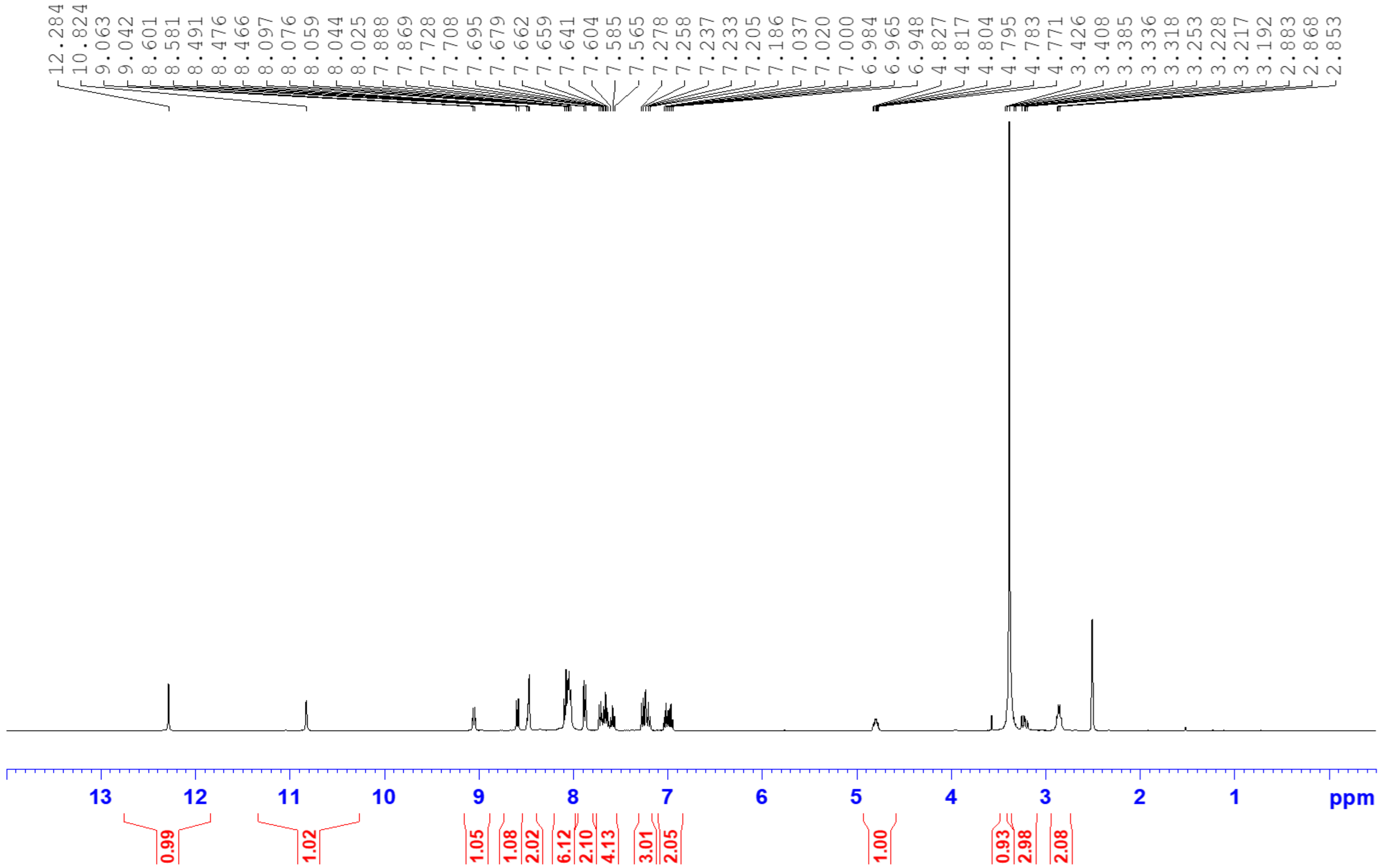

^{13}C NMR spectra of (*S*)-*N*-(2-((1-((2-aminoethyl)amino)-3-(1*H*-indol-3-yl)-1-oxopropan-2-yl)carbamoyl)phenyl)-2-naphthamide (**1**)

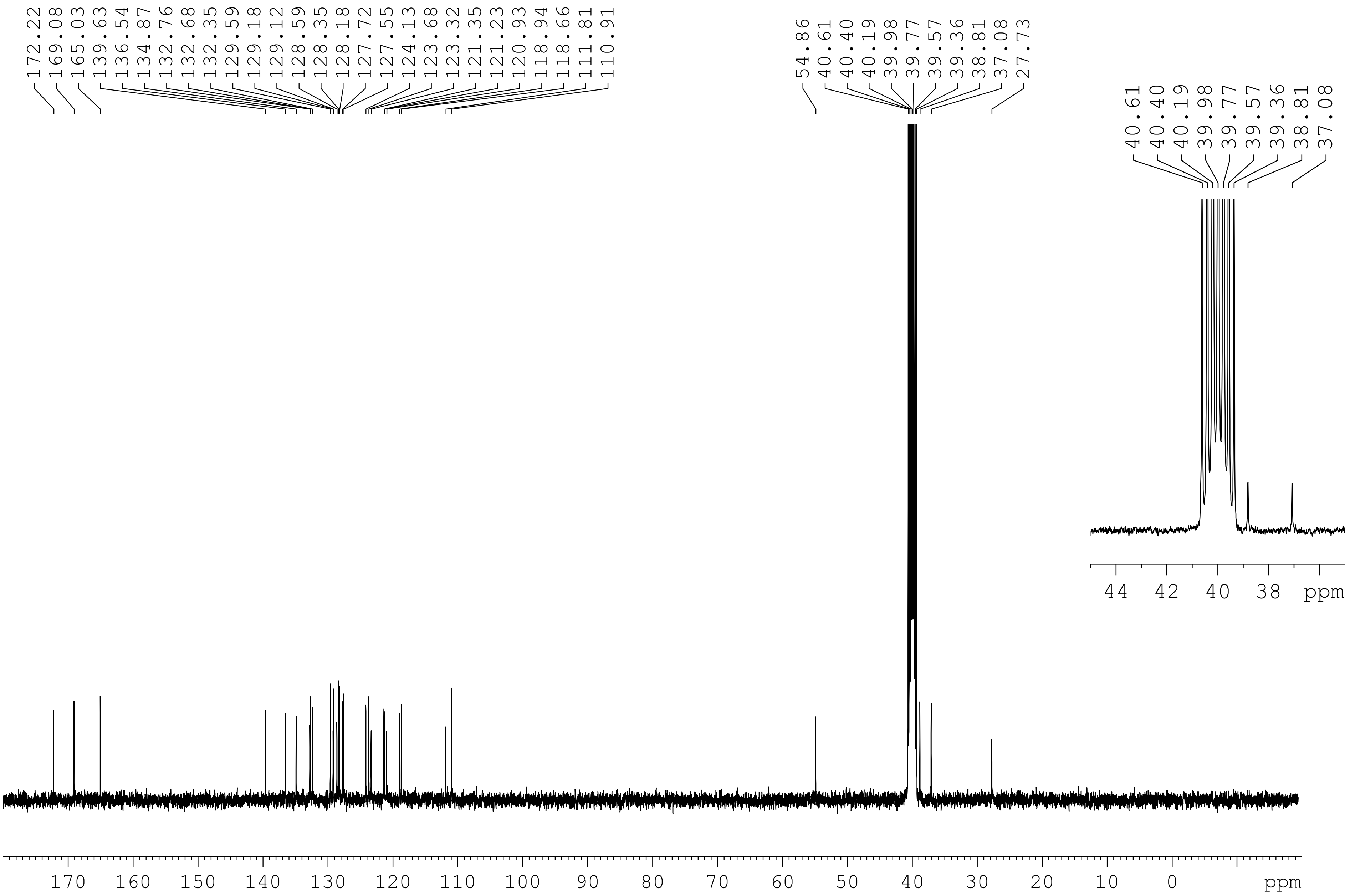

^{1}H NMR spectra of (*S*)-*N*-(2-((1-((2-aminoethyl)amino)-3-(*1H*-indol-3-yl)-1-oxopropan-2-yl)carbamoyl)-4-fluorophenyl)-2-naphthamide (**2**)

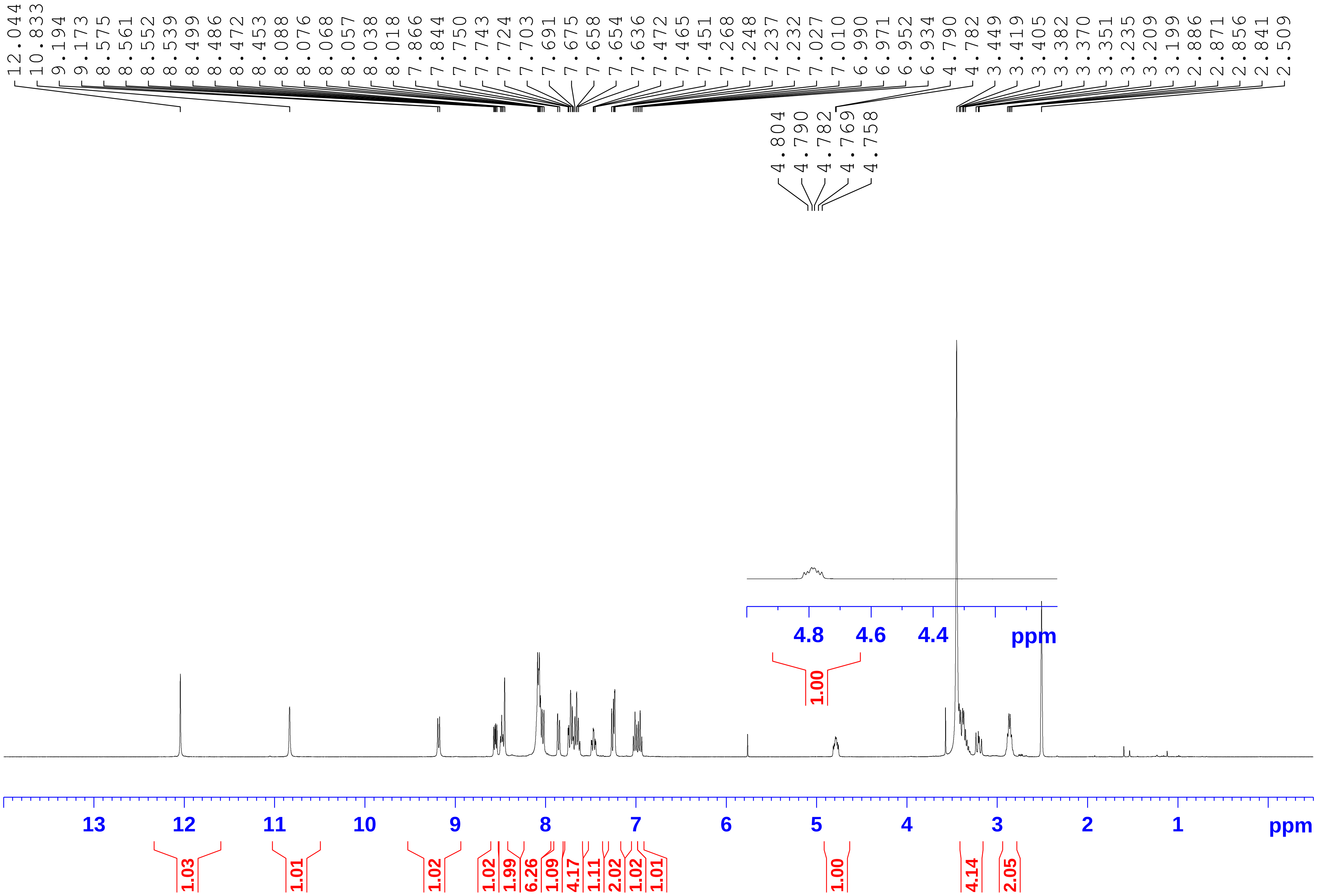
12.044
10.833
9.194
9.173
8.575
8.561
8.552
8.539
8.499
8.486
8.472
8.453
8.088
8.076
8.068
8.057
8.038
8.018
7.866
7.844
7.750
7.743
7.724
7.703
7.691
7.675
7.658
7.654
7.636
7.472
7.465
7.451
7.268
7.248
7.237
7.232
7.027
7.010
6.990
6.971
6.952
6.934
4.790
4.782
3.449
3.419
3.405
3.382
3.370
3.351
3.235
3.209
3.199
2.886
2.871
2.856
2.841
2.509
4.804
4.790
4.782
4.769
4.758
4.8
4.6
4.4
ppm
1.00
13
12
11
10
9
8
7
6
5
4
3
2
1
ppm
1.03
1.01
1.02
1.02
1.99
6.26
1.09
4.17
1.11
2.02
1.02
1.01
1.00
4.14
2.05

^{13}C NMR spectra of (*S*)-*N*-(2-((1-((2-aminoethyl)amino)-3-(*1H*-indol-3-yl)-1-oxopropan-2-yl)carbamoyl)-4-fluorophenyl)-2-naphthamide (**2**)

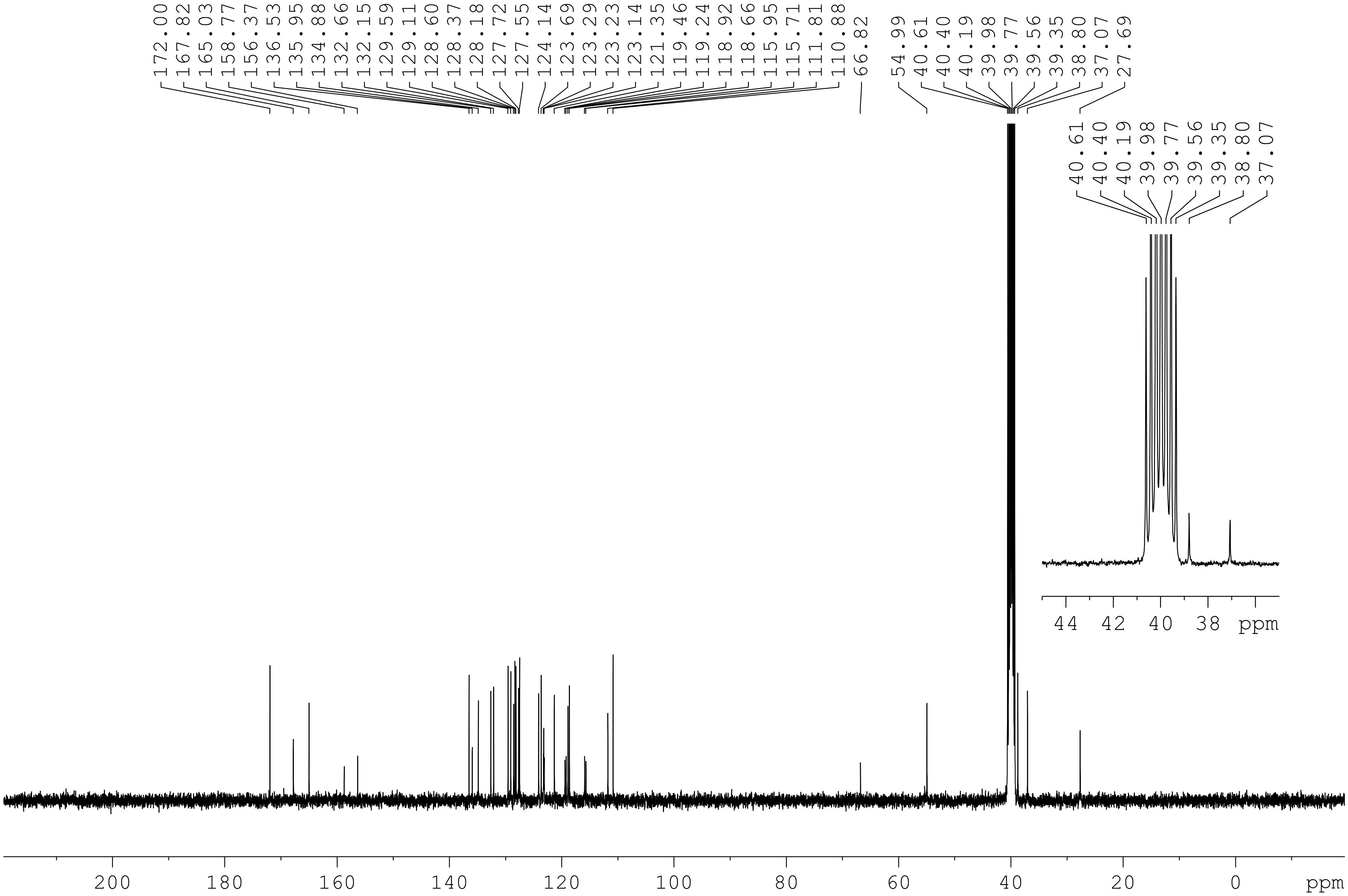

^{1}H NMR spectra of (*S*)-*N*-(2-((1-((2-aminoethyl)amino)-3-(*1H*-indol-3-yl)-1-oxopropan-2-yl)carbamoyl)-4-chlorophenyl)-2-naphthamide (**3**)

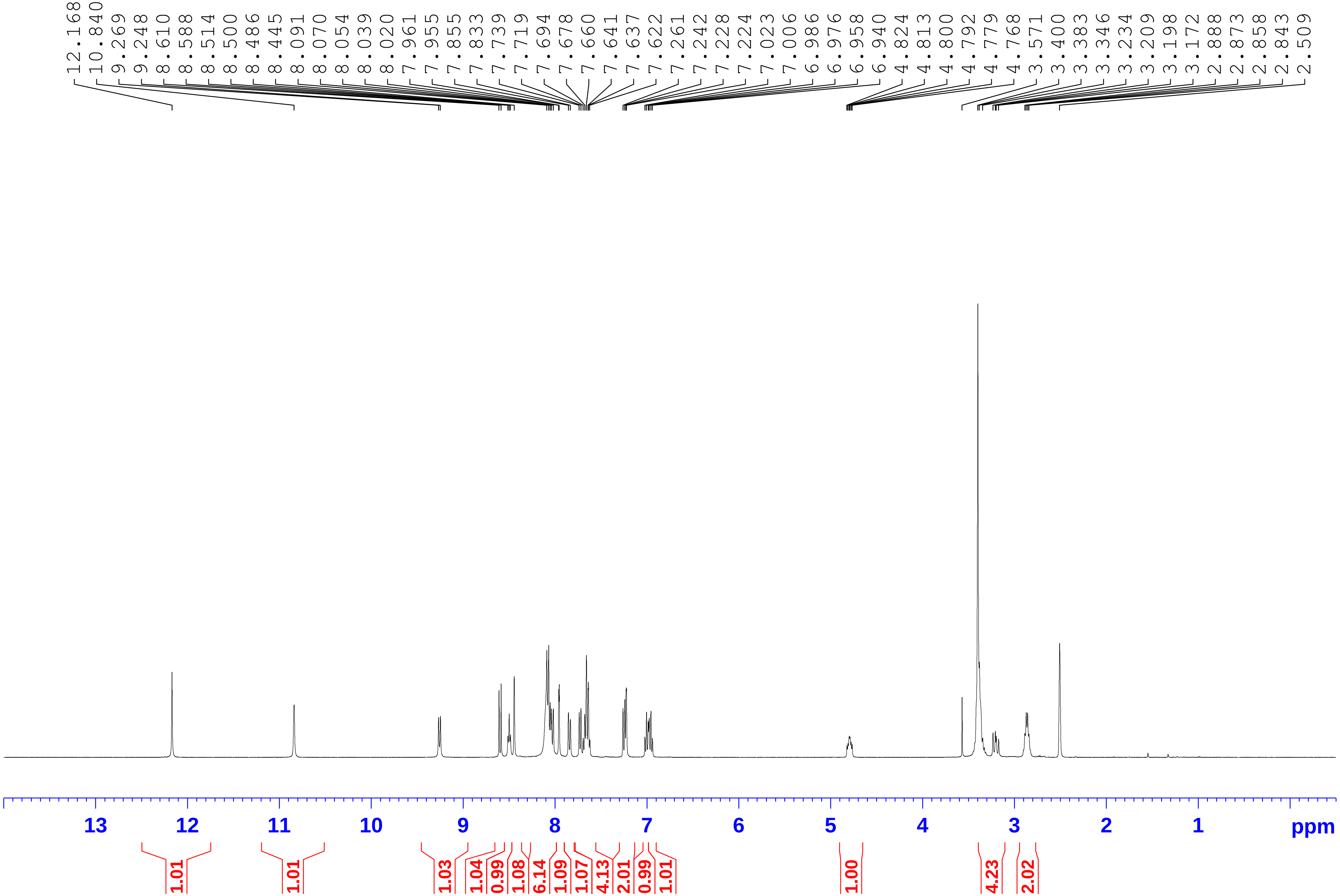

^{13}C NMR spectra of (*S*)-*N*-(2-((1-((2-aminoethyl)amino)-3-(*1H*-indol-3-yl)-1-oxopropan-2-yl)carbamoyl)-4-chlorophenyl)-2-naphthamide (**3**)

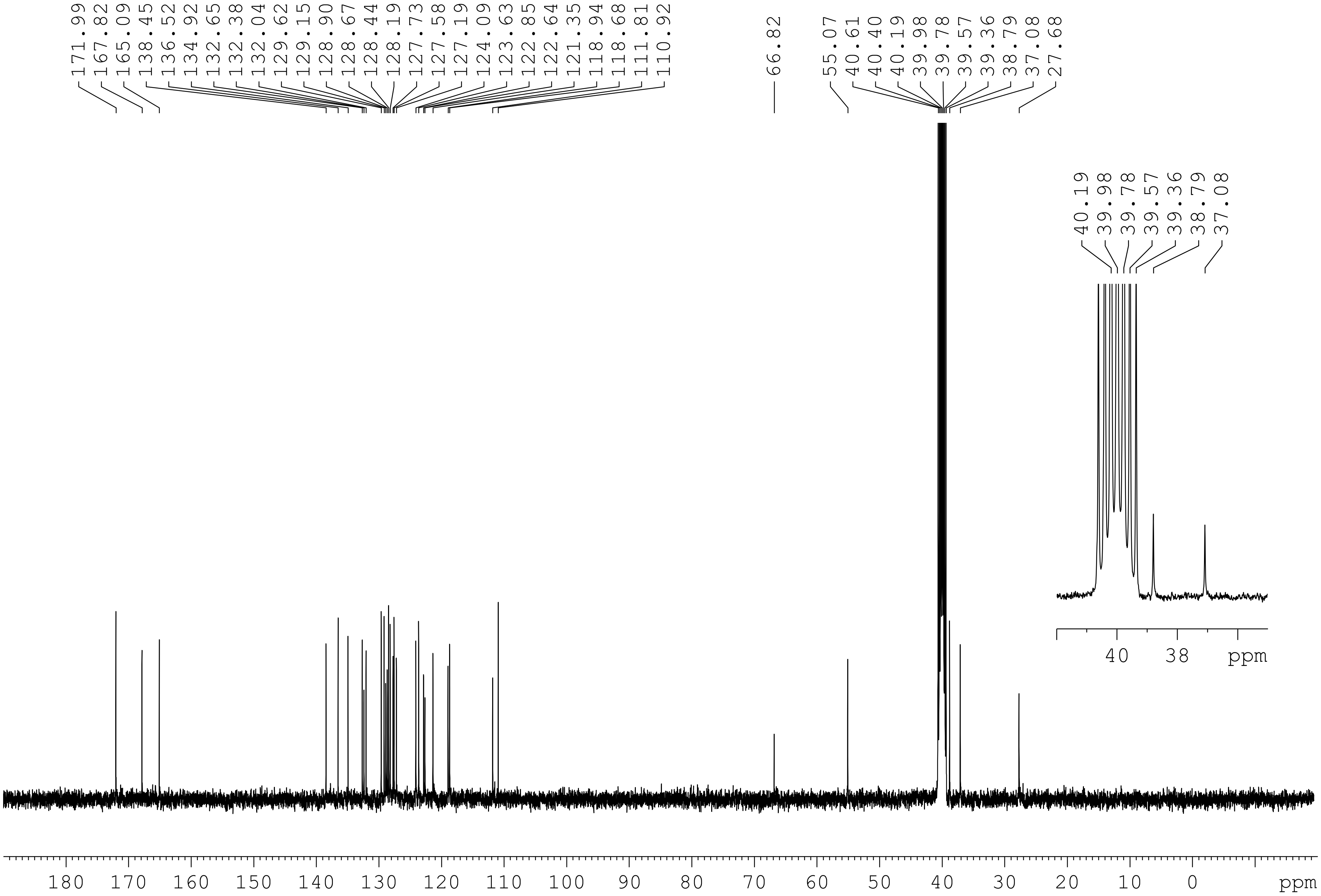

^{1}H NMR spectra of (*S*)-*N*-(2-((1-((2-aminoethyl)amino)-3-(*1H*-indol-3-yl)-1-oxopropan-2-yl)carbamoyl)-4-bromophenyl)-2-naphthamide (**4**)

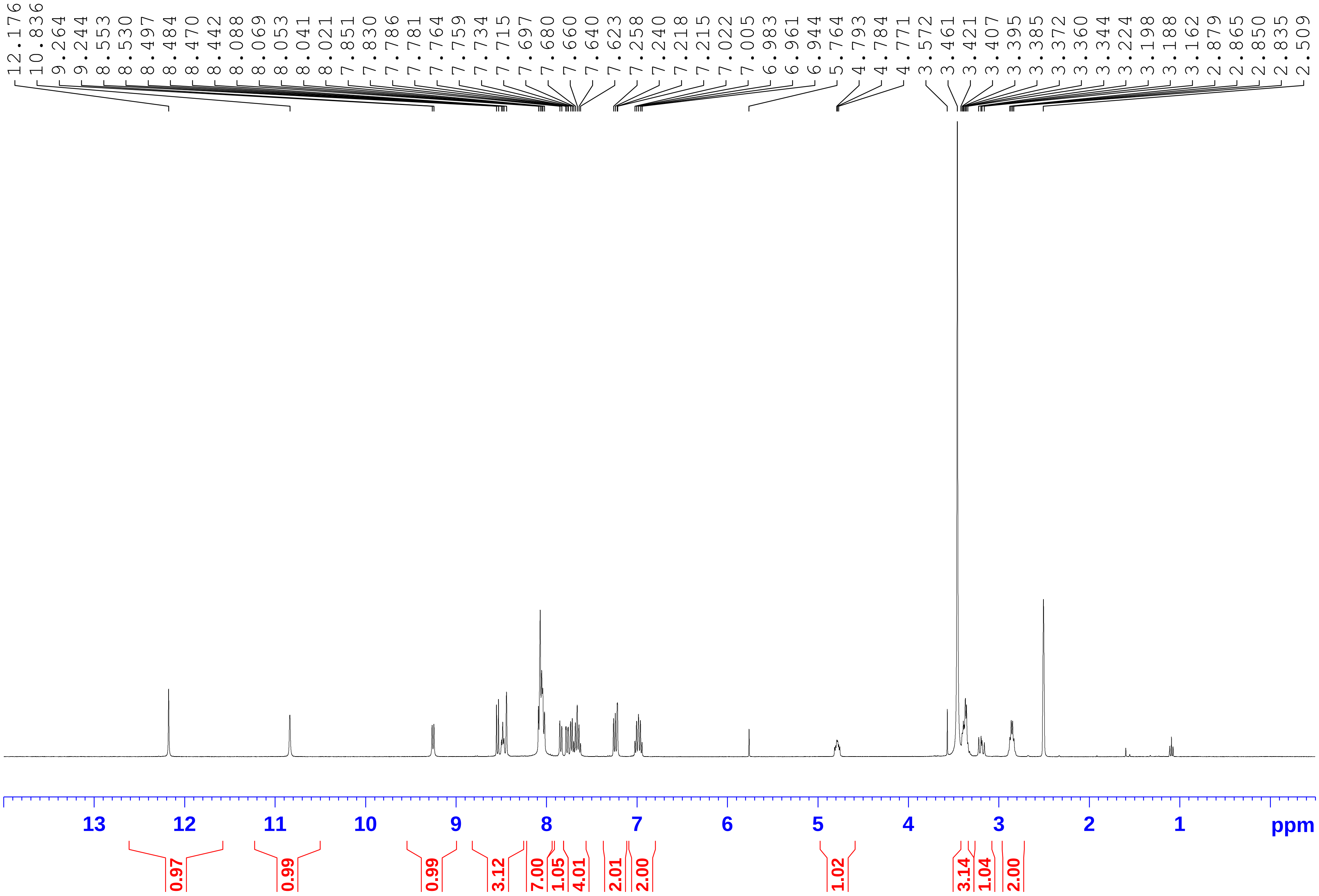

^{13}C NMR spectra of (*S*)-*N*-(2-((1-((2-aminoethyl)amino)-3-(1*H*-indol-3-yl)-1-oxopropan-2-yl)carbamoyl)-4-bromophenyl)-2-naphthamide (**4**)

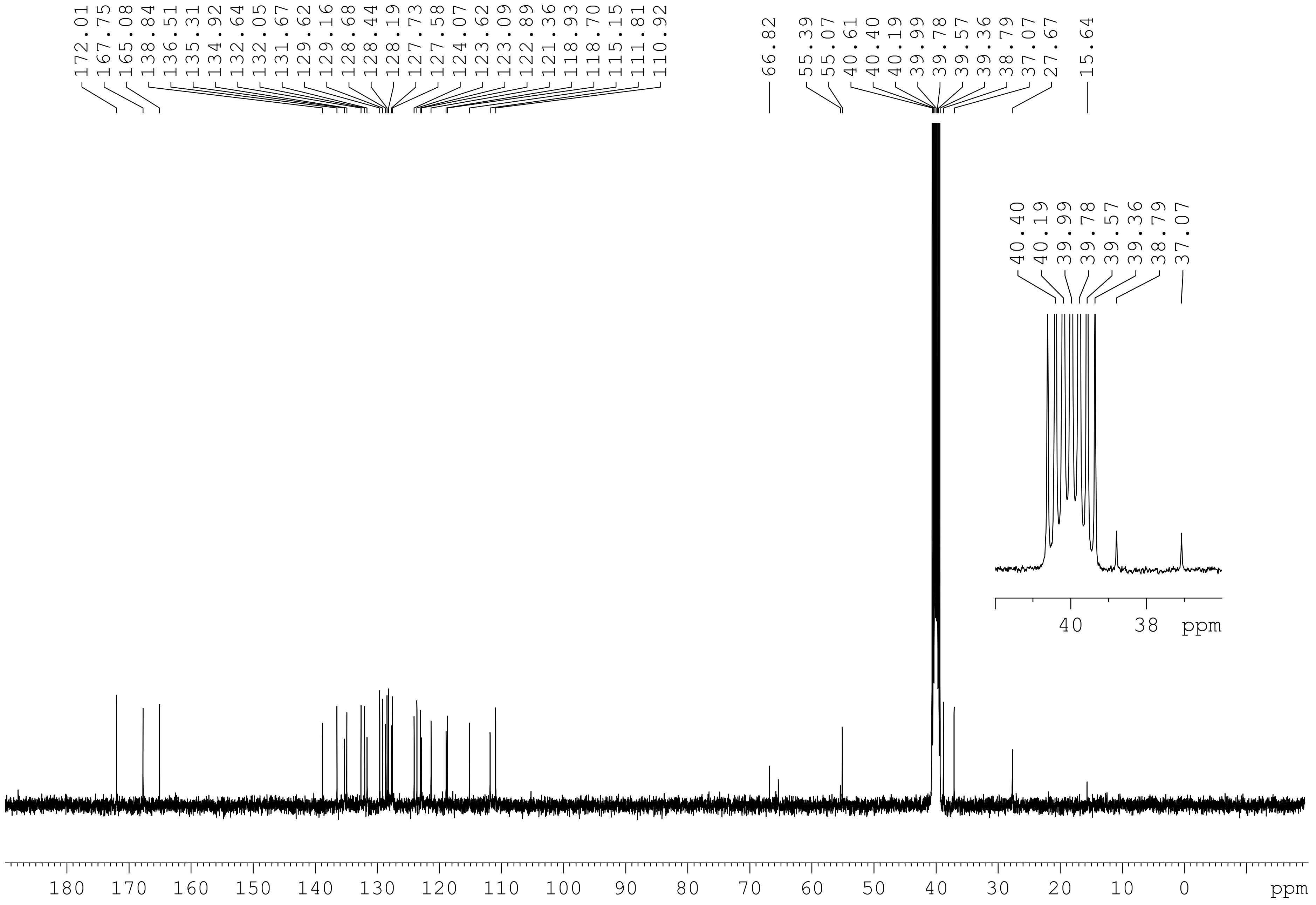

^{1}H NMR spectra of (*S*)-*N*-(2-((1-((2-aminoethyl)amino)-3-(*1H*-indol-3-yl)-1-oxopropan-2-yl)carbamoyl)-4-methoxyphenyl)-2-naphthamide (**5**)

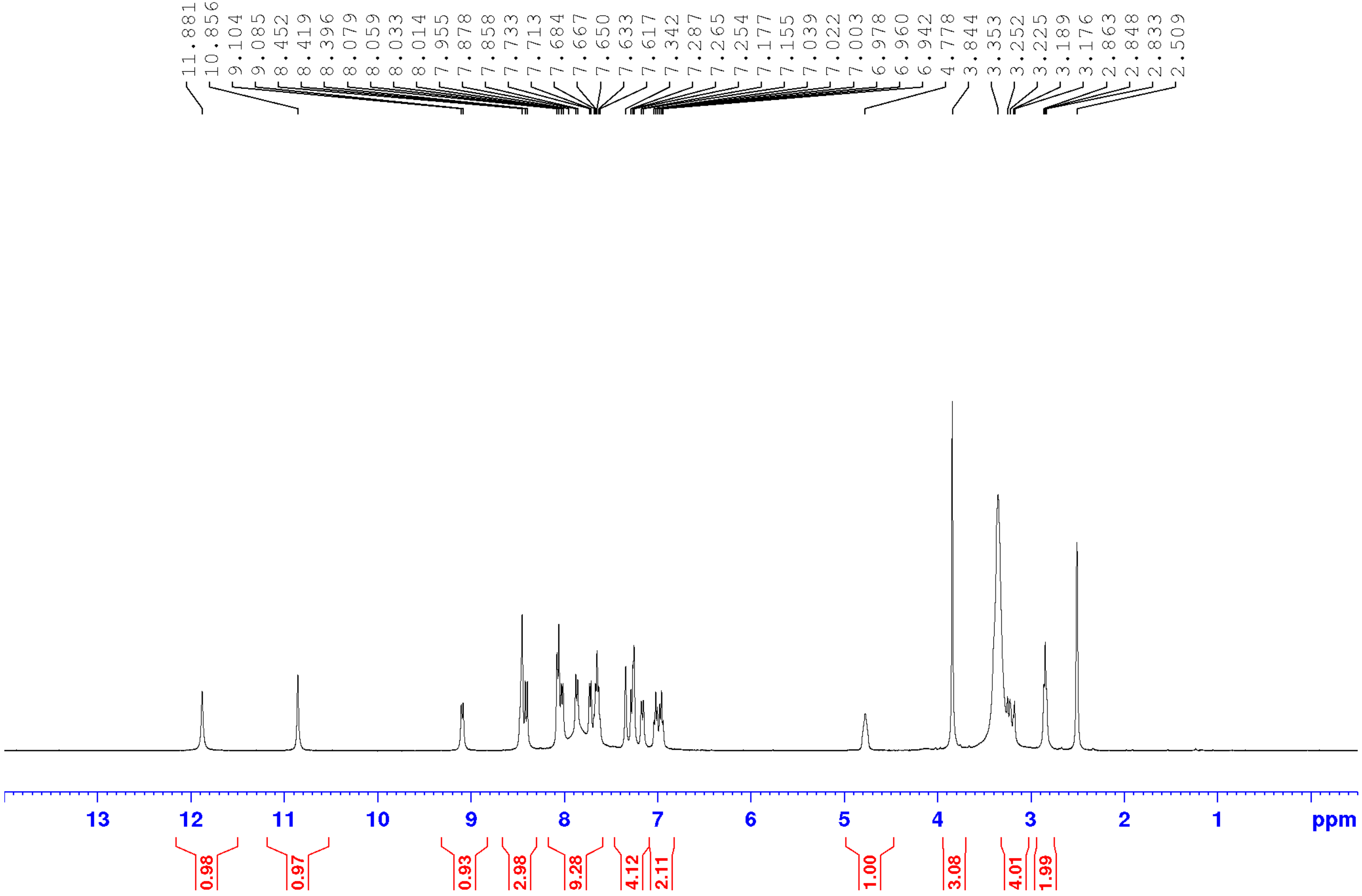

^{13}C NMR spectra of (*S*)-*N*-(2-((1-((2-aminoethyl)amino)-3-(*1H*-indol-3-yl)-1-oxopropan-2-yl)carbamoyl)-4-methoxyphenyl)-2-naphthamide (**5**)

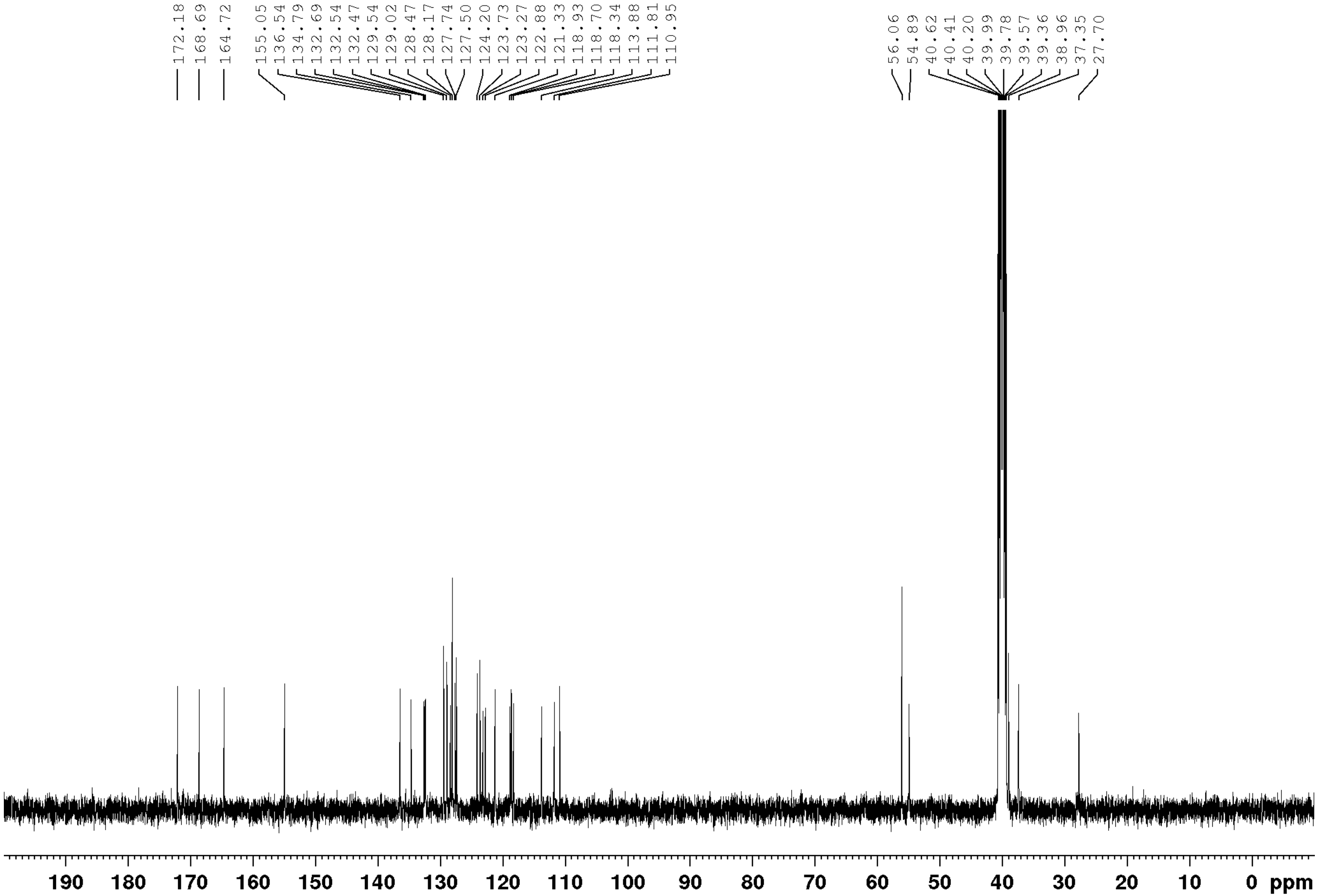

^{1}H NMR spectra of (*S*)-*N*-(2-((1-((2-guanidinoethyl)amino)-3-(*1H*-indol-3-yl)-1-oxopropan-2-yl)carbamoyl)phenyl)-2-naphthamide (**6**)

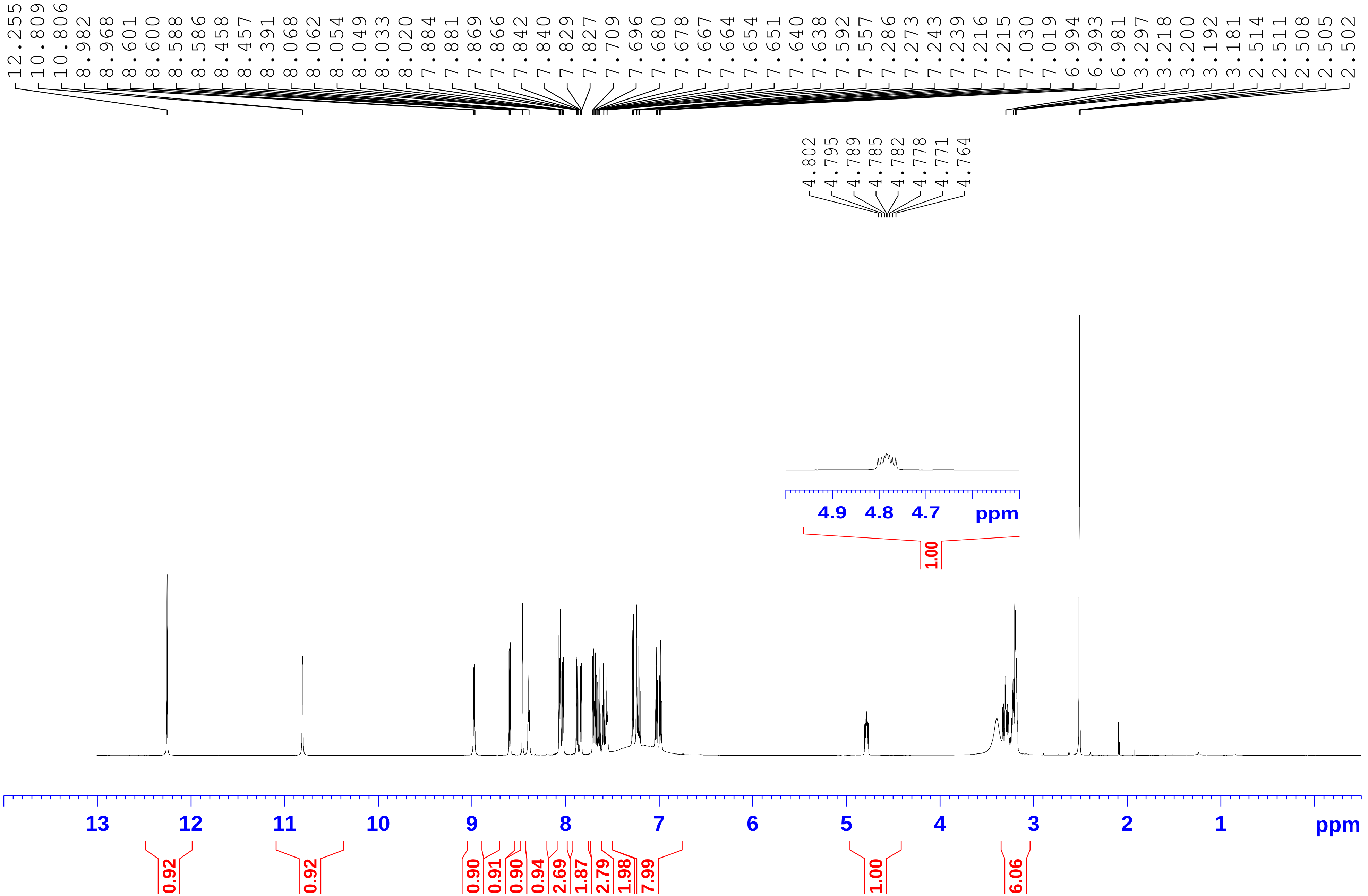

^{13}C NMR spectra of (*S*)-*N*-(2-((1-((2-guanidinoethyl)amino)-3-(*1H*-indol-3-yl)-1-oxopropan-2-yl)carbamoyl)phenyl)-2-naphthamide (**6**)

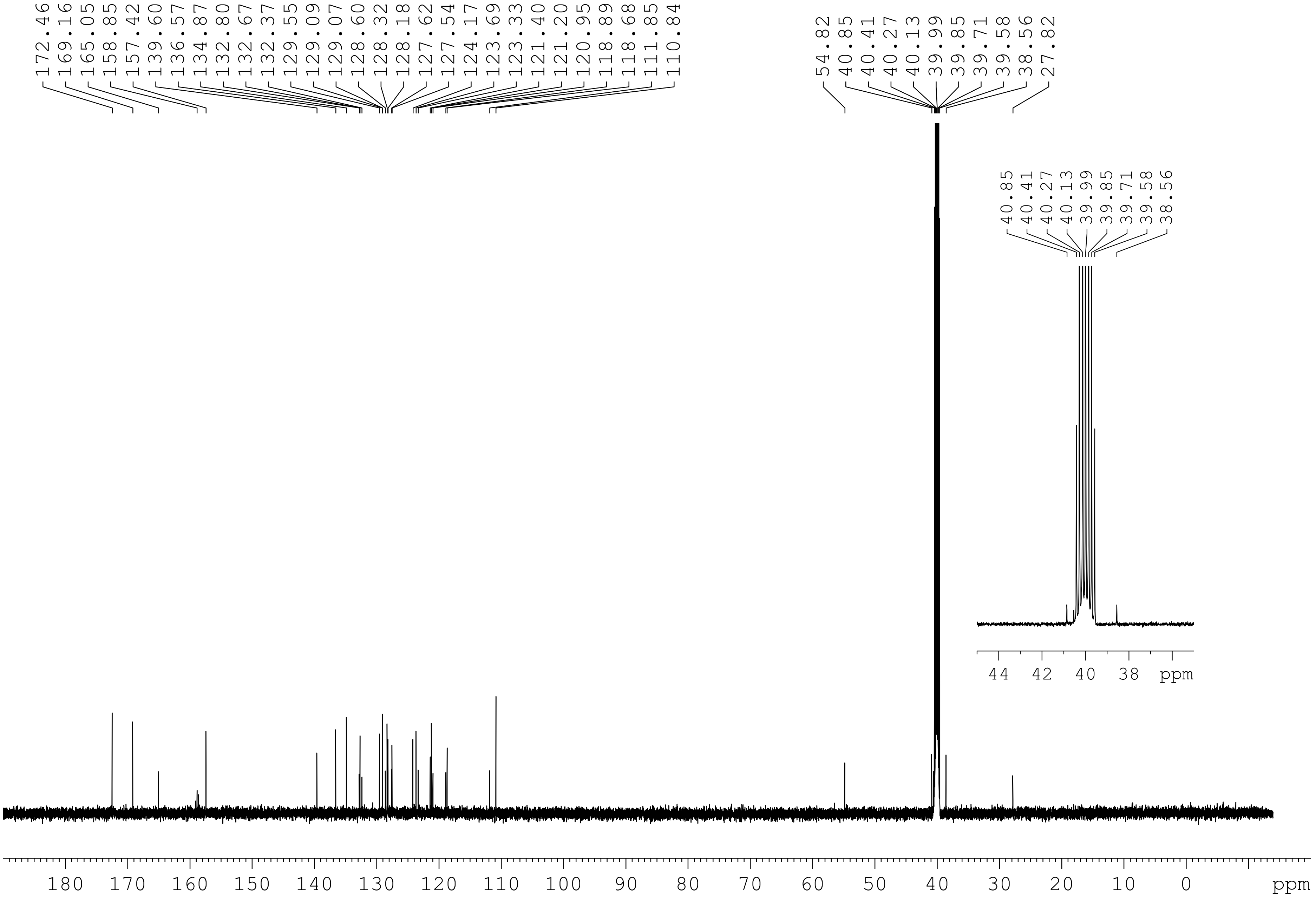

^{1}H NMR spectra of (*S*)-*N*-(4-fluoro-2-((1-((2-guanidinoethyl)amino)-3-(*1H*-indol-3-yl)-1-oxopropan-2-yl)carbamoyl)phenyl)-2-naphthamide (**7**)

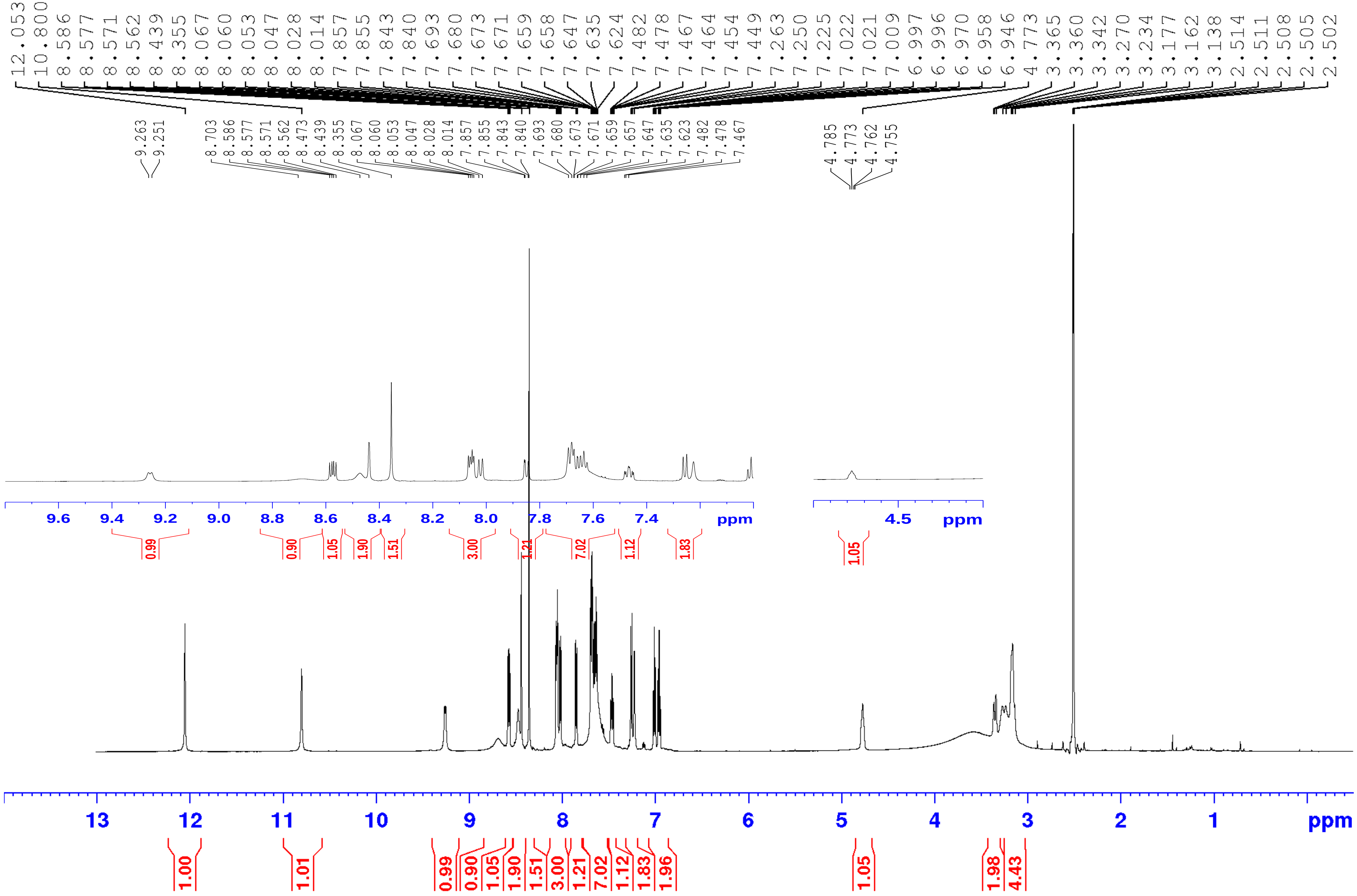

^{13}C NMR spectra of (*S*)-*N*-(4-fluoro-2-((1-((2-guanidinoethyl)amino)-3-(*1H*-indol-3-yl)-1-oxopropan-2-yl)carbamoyl)phenyl)-2-naphthamide (**7**)

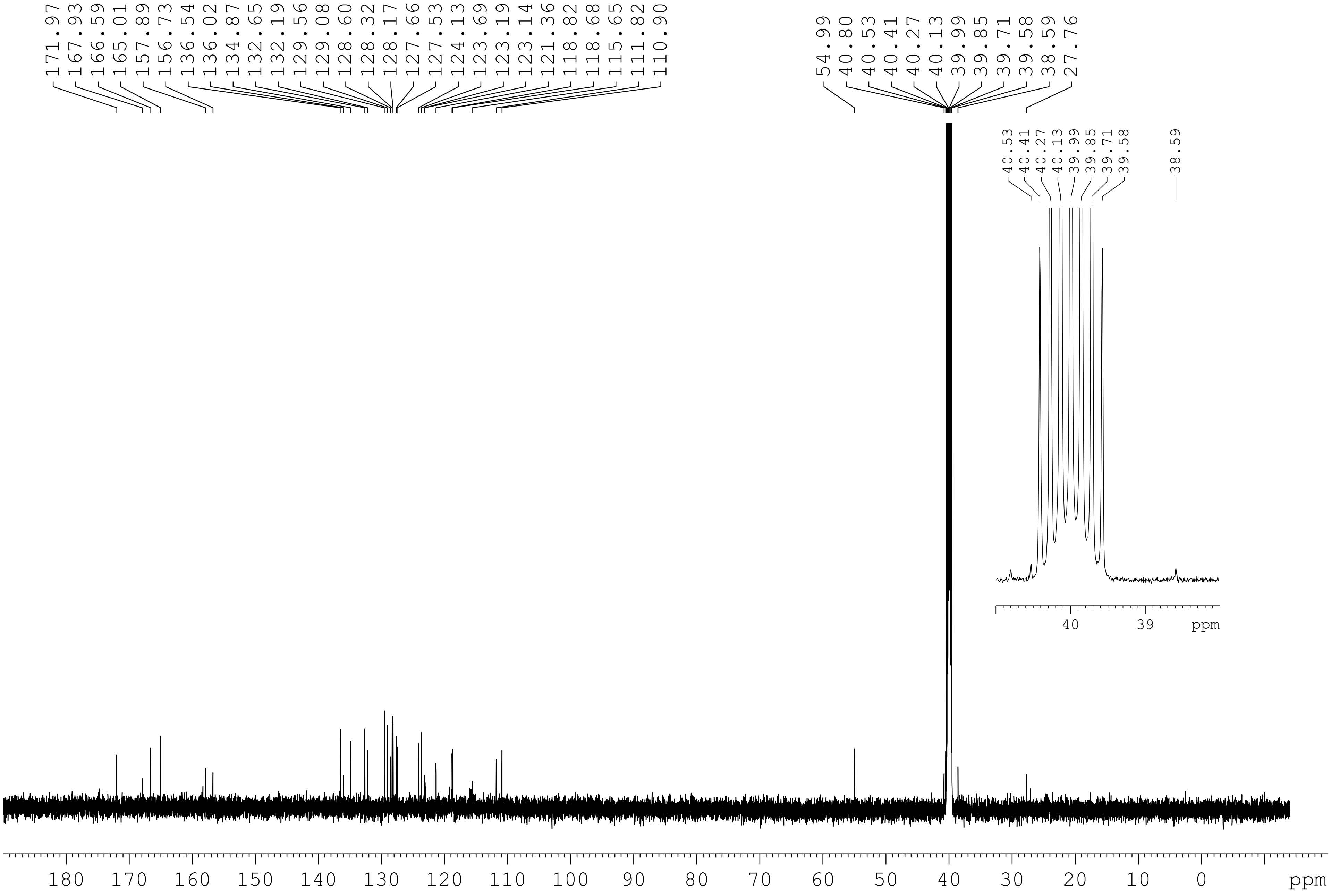

^{1}H NMR spectra of (*S*)-*N*-(4-chloro-2-((1-((2-guanidinoethyl)amino)-3-(*1H*-indol-3-yl)-1-oxopropan-2-yl)carbamoyl)phenyl)-2-naphthamide (**8**)

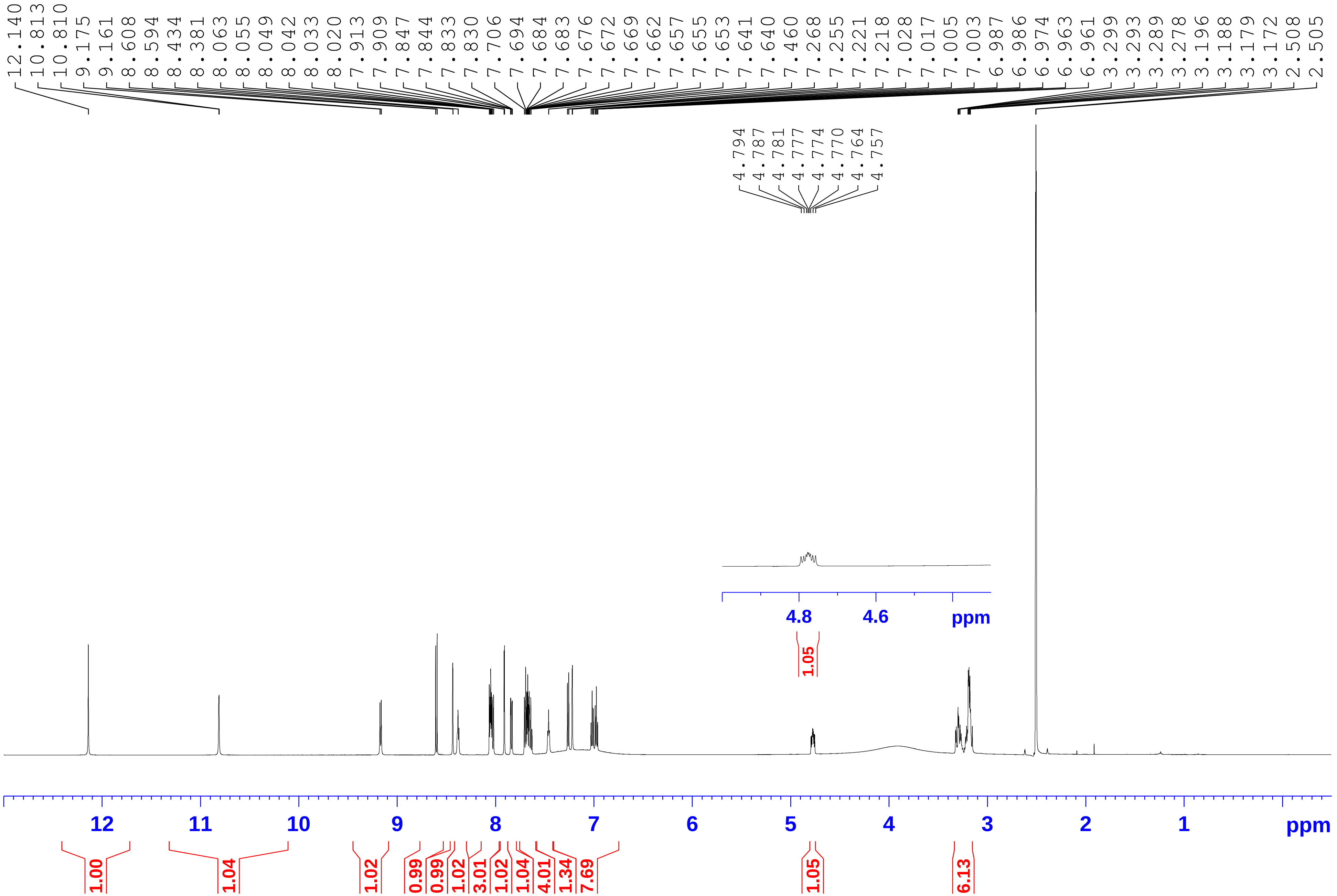

^{13}C NMR spectra of (*S*)-*N*-(4-chloro-2-((1-((2-guanidinoethyl)amino)-3-(*1H*-indol-3-yl)-1-oxopropan-2-yl)carbamoyl)phenyl)-2-naphthamide (**8**)

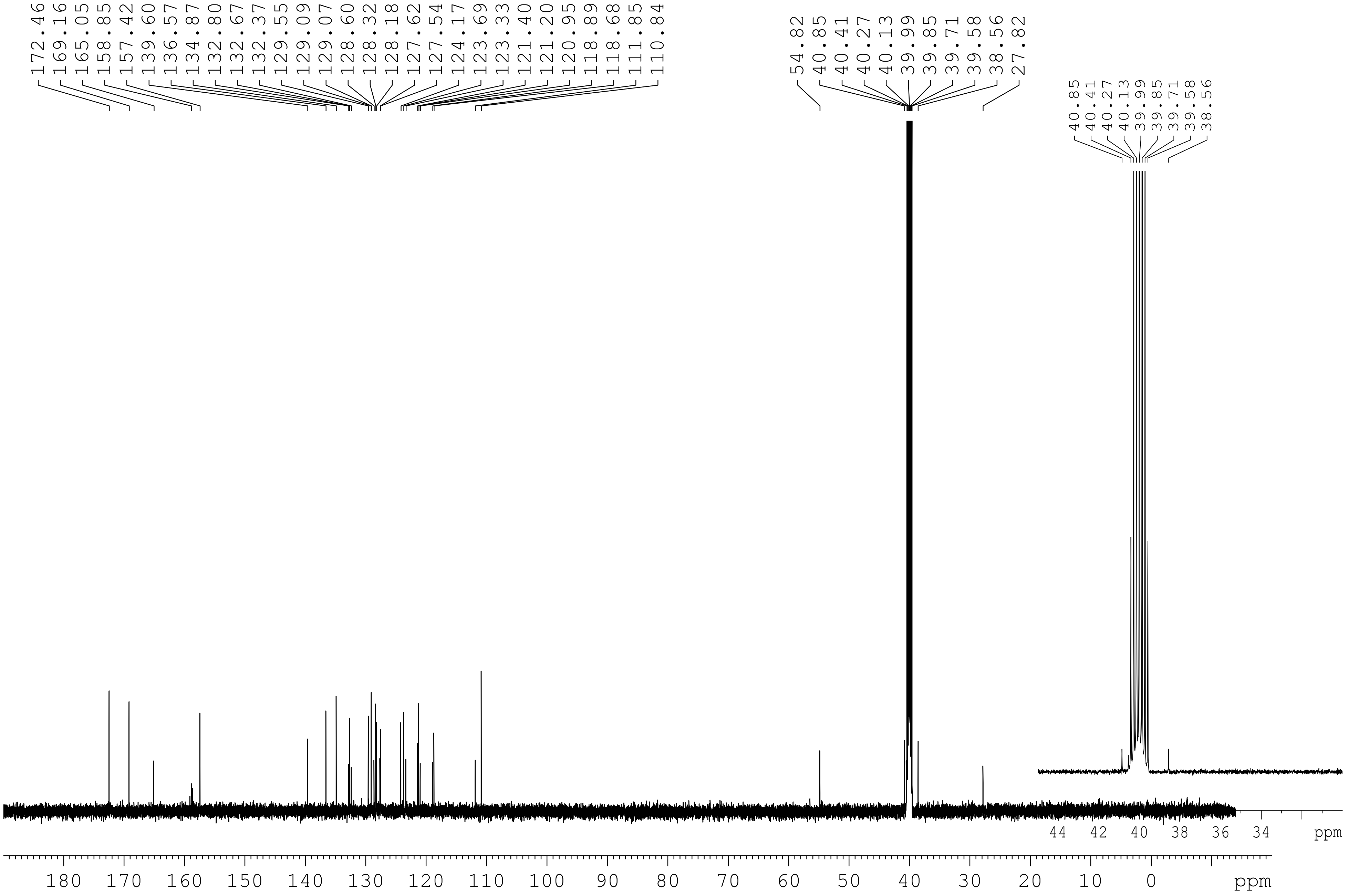

^{1}H NMR spectra of (*S*)-*N*-(4-bromo-2-((1-((2-guanidinoethyl)amino)-3-(1*H*-indol-3-yl)-1-oxopropan-2-yl)carbamoyl)phenyl)-2-naphthamide (**9**)

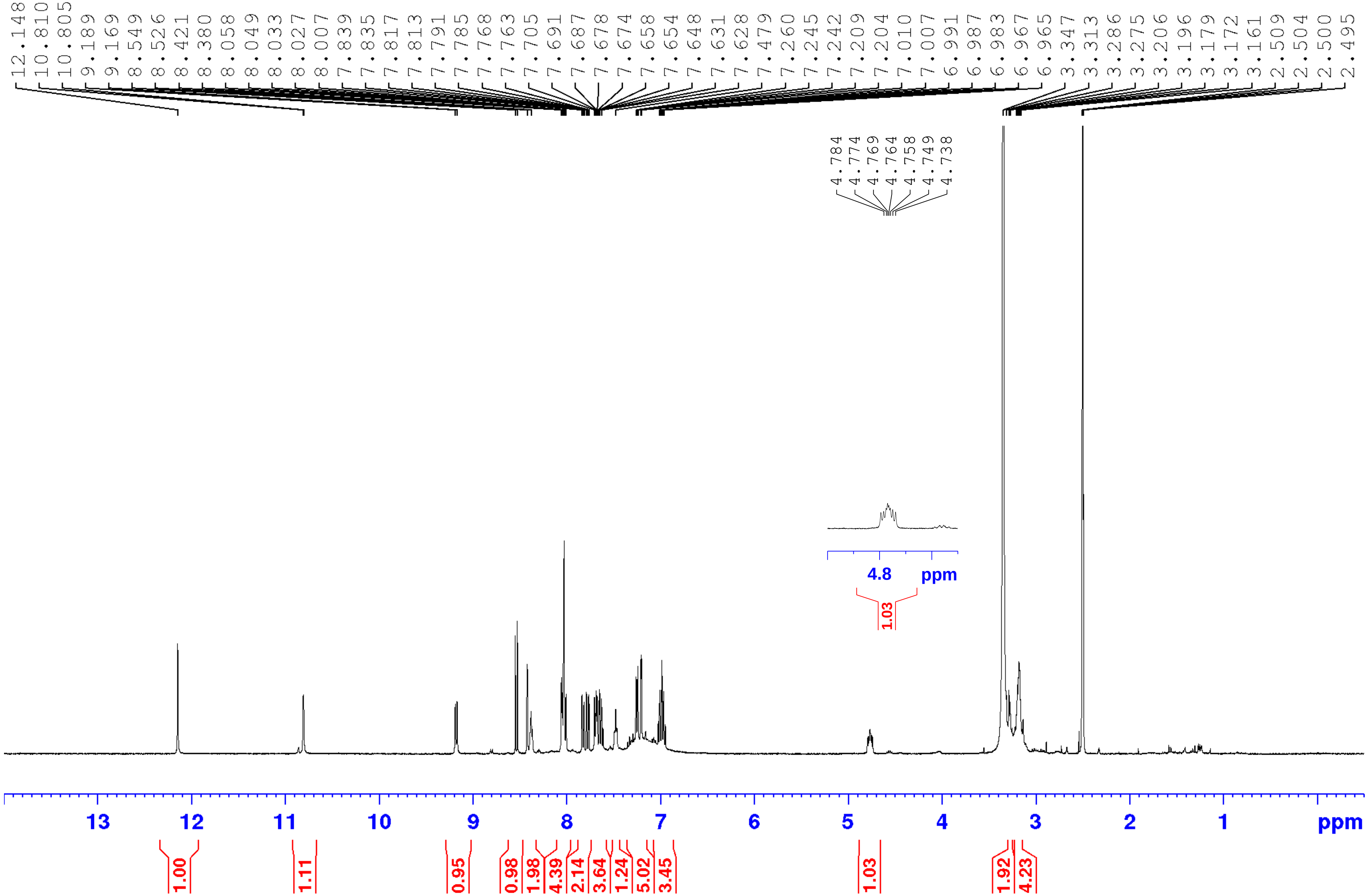

^{13}C NMR spectra of (*S*)-*N*-(4-bromo-2-((1-((2-guanidinoethyl)amino)-3-(1*H*-indol-3-yl)-1-oxopropan-2-yl)carbamoyl)phenyl)-2-naphthamide (**9**)

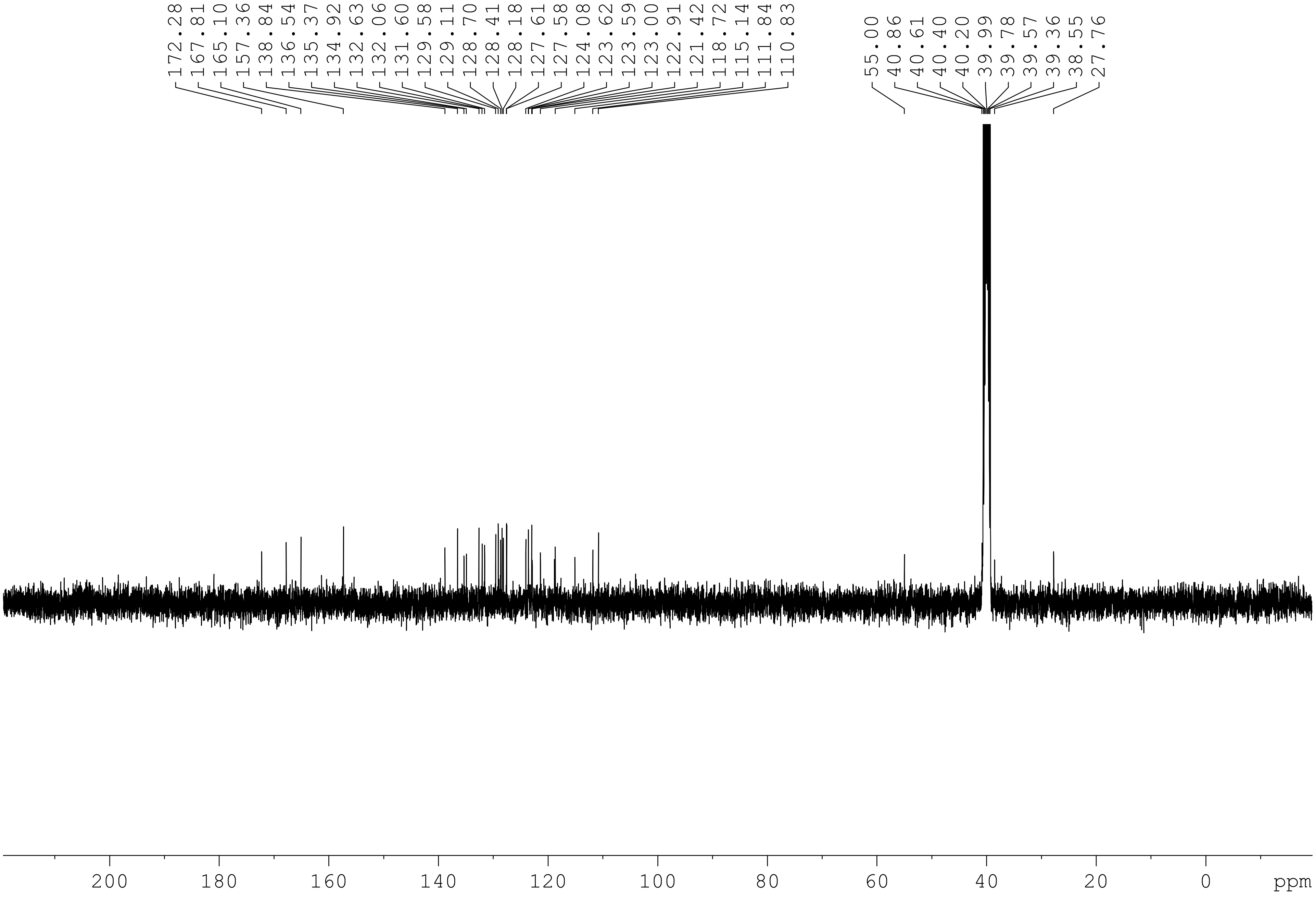

^{1}H NMR spectra of (*S*)-*N*-(2-((1-((2-guanidinoethyl)amino)-3-(*1H*-indol-3-yl)-1-oxopropan-2-yl)carbamoyl)-4-methoxyphenyl)-2-naphthamide (**10**)

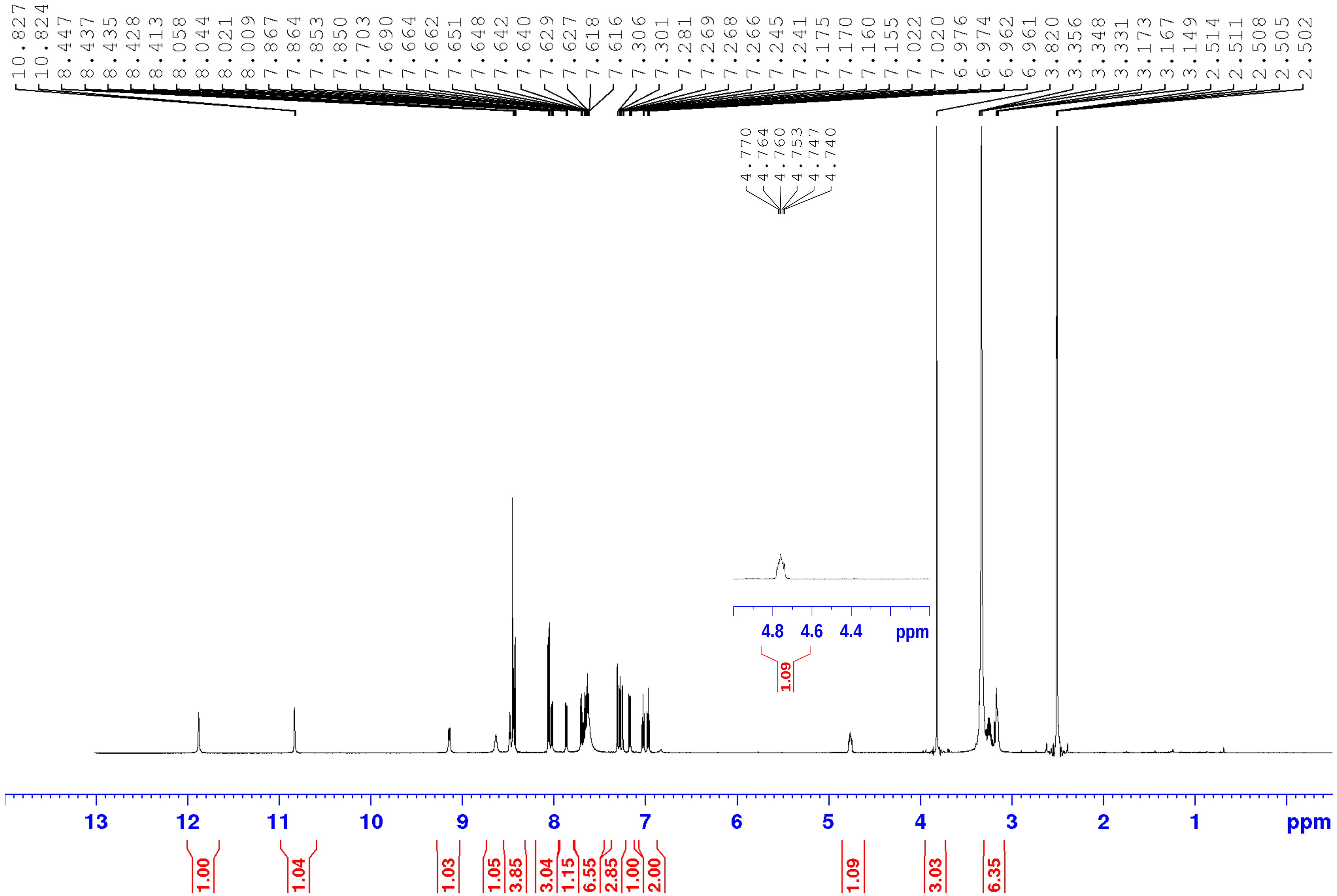

^{13}C NMR spectra of (*S*)-*N*-(2-((1-((2-guanidinoethyl)amino)-3-(*1H*-indol-3-yl)-1-oxopropan-2-yl)carbamoyl)-4-methoxyphenyl)-2-naphthamide (**10**)

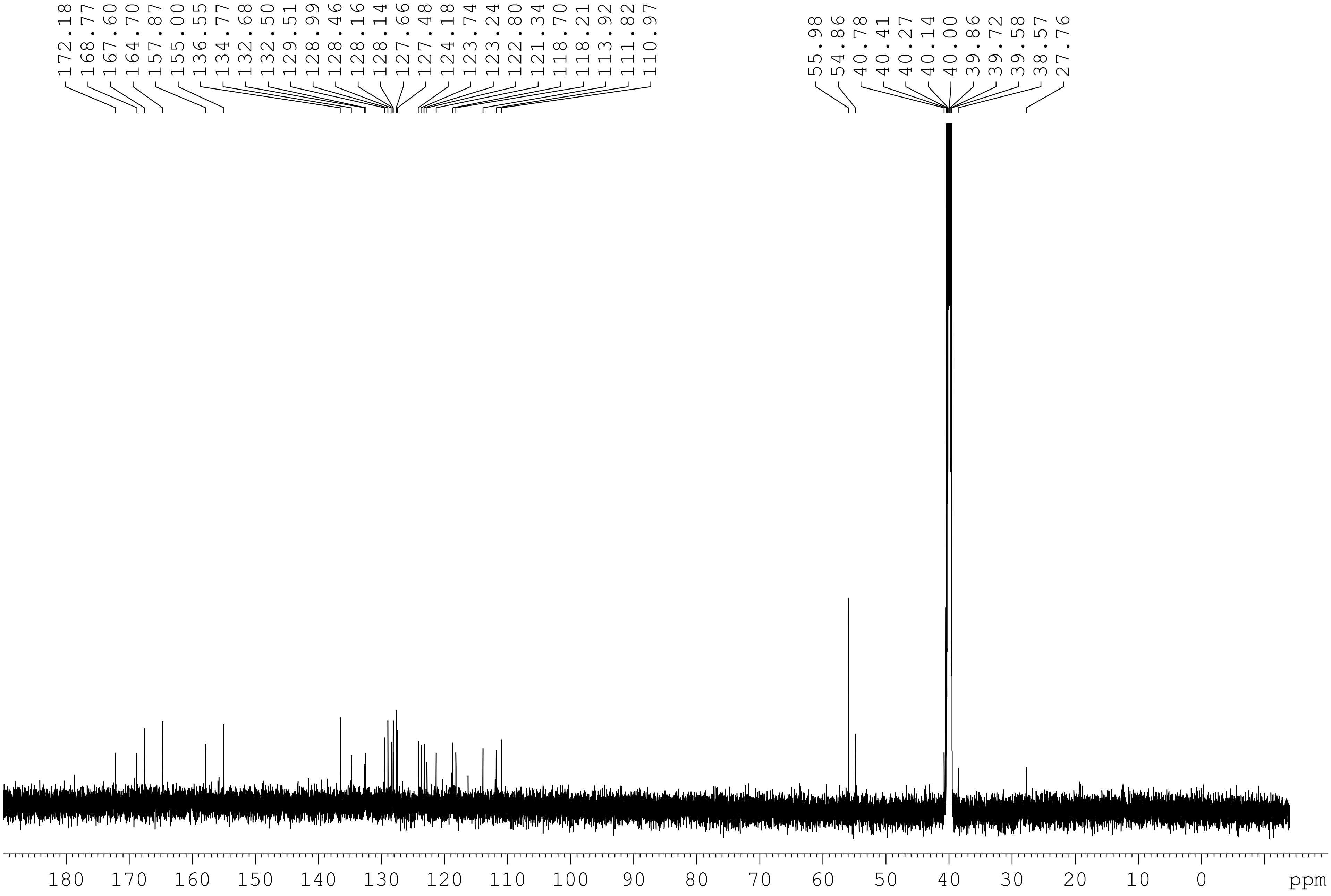

^{1}H NMR of (*S*)-2-amino-*N*-(1-((2-aminoethyl)amino)-3-(1*H*-indol-3-yl)-1-oxopropan-2-yl)-5-(naphthalen-2-yl)benzamide (**11**)

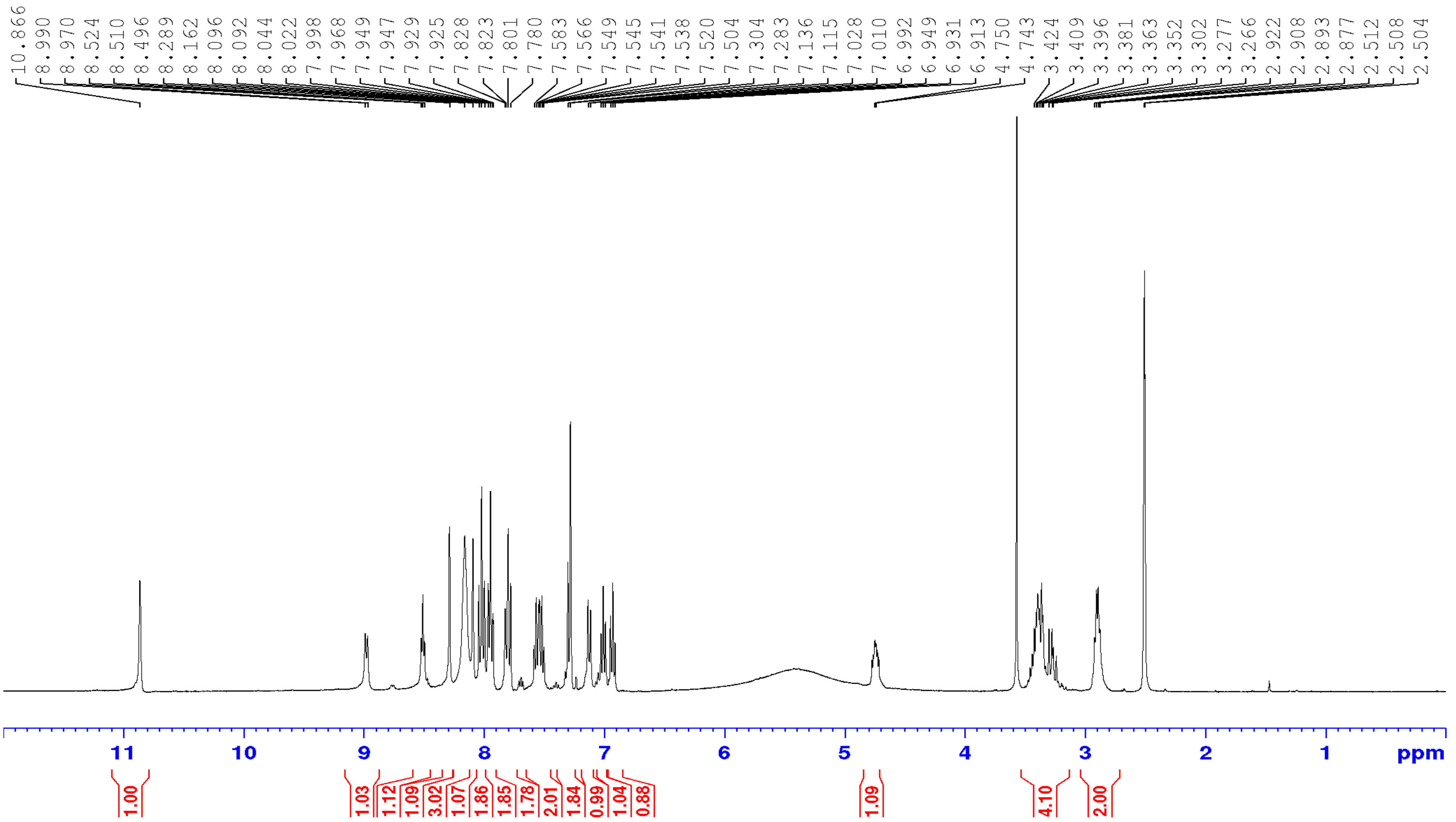

^{13}C NMR of (*S*)-2-amino-*N*-(1-((2-aminoethyl)amino)-3-(1*H*-indol-3-yl)-1-oxopropan-2-yl)-5-(naphthalen-2-yl)benzamide (**11**)

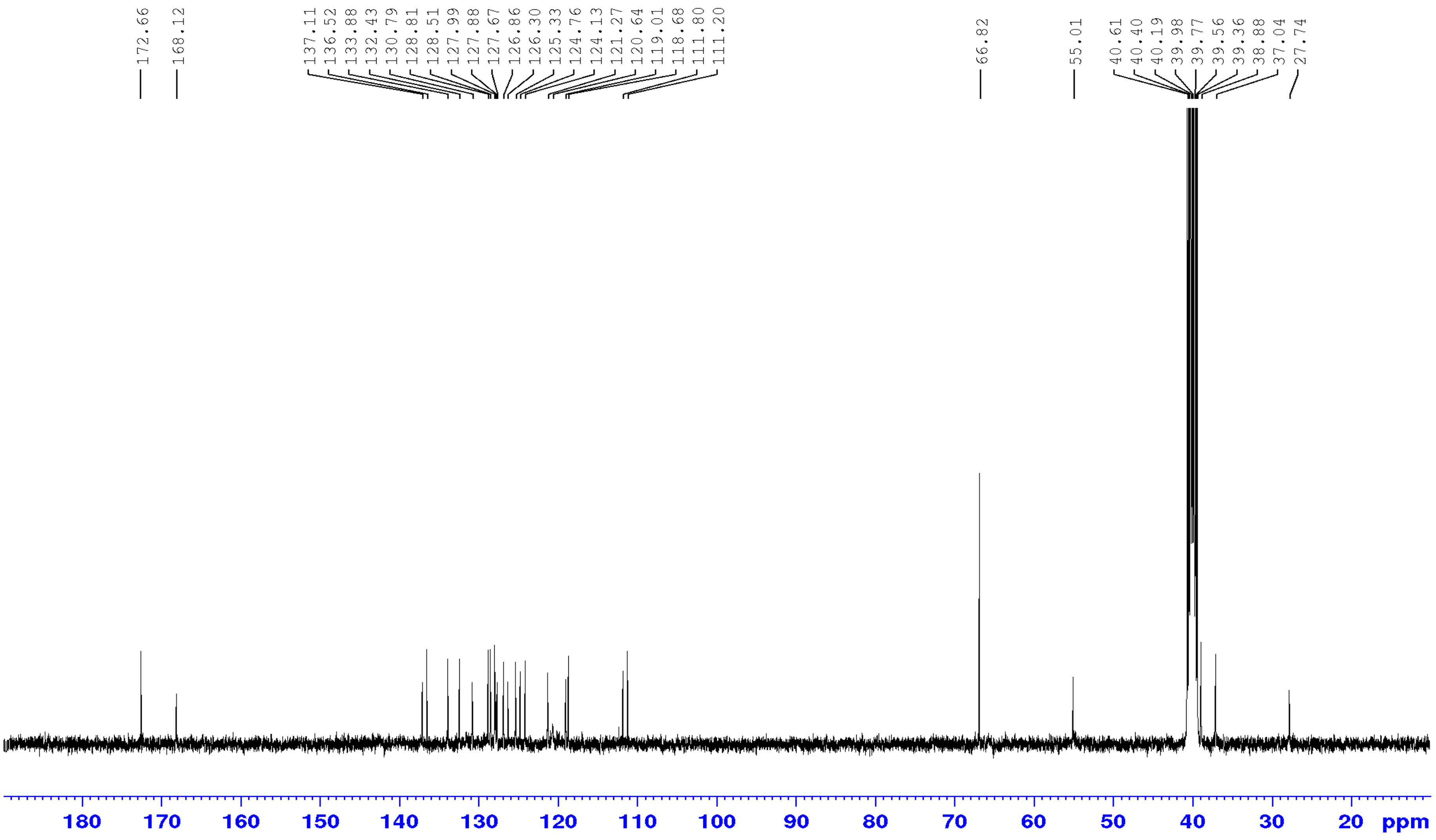

^{1}H NMR of (*S*)-3-((1-((2-ammonioethyl)amino)-3-(1*H*-indol-3-yl)-1-oxopropan-2-yl)carbamoyl)-[1,1':4',1"-terphenyl]-4-aminium (**12**)

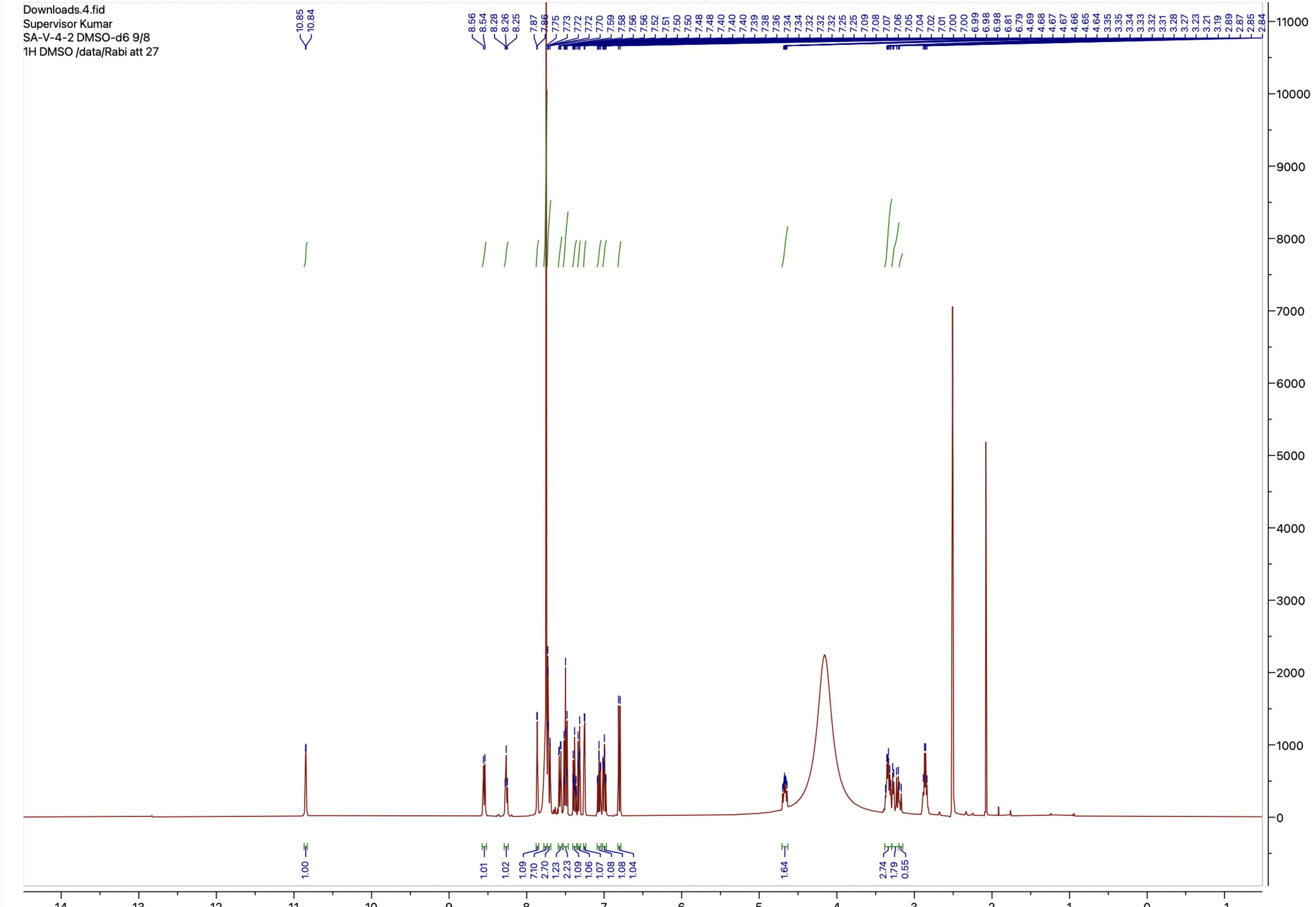

^{13}C NMR of (*S*)-3-((1-((2-ammonioethyl)amino)-3-(1*H*-indol-3-yl)-1-oxopropan-2-yl)carbamoyl)-[1,1':4',1''-terphenyl]-4-aminium (**12**)

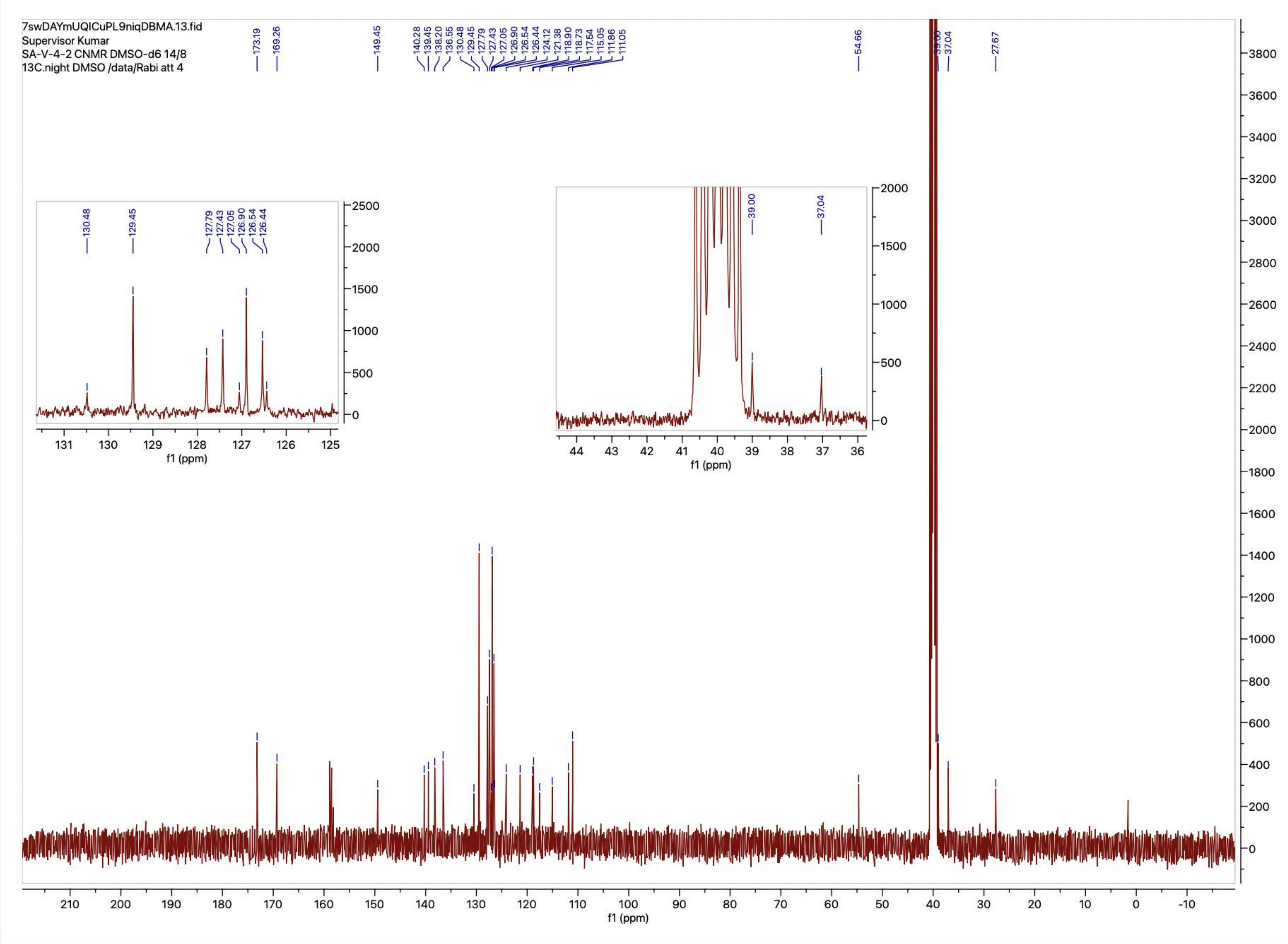

^{1}H NMR of (*S*)-2-((1-((2-ammonioethyl)amino)-3-(1*H*-indol-3-yl)-1-oxopropan-2-yl)carbamoyl)-4-(2,4-dimethoxypyrimidin-5-yl)benzenaminium (**13**)

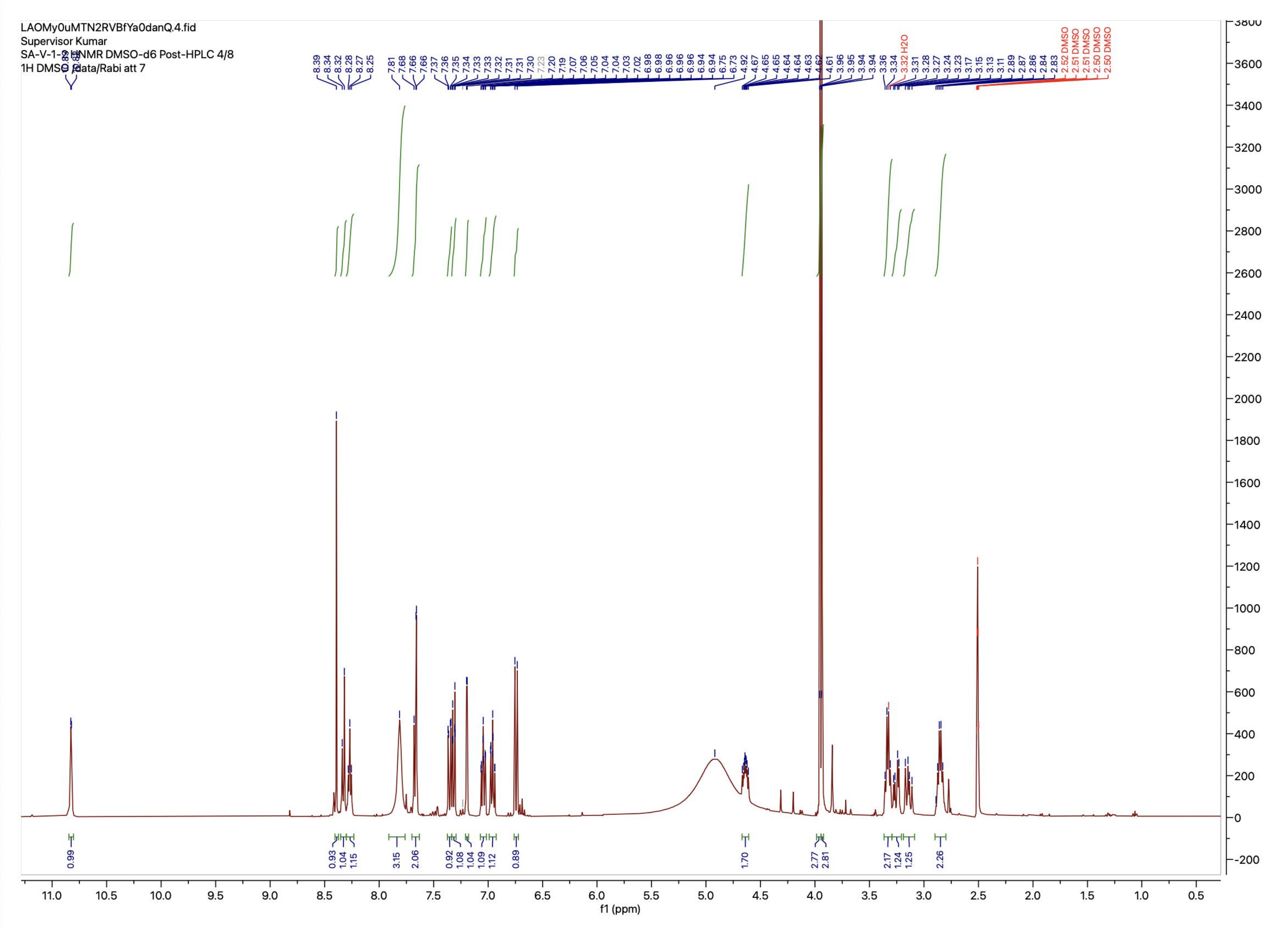

^{13}C NMR of (*S*)-2-((1-((2-ammonioethyl)amino)-3-(1*H*-indol-3-yl)-1-oxopropan-2-yl)carbamoyl)-4-(2,4-dimethoxypyrimidin-5-yl)benzenaminium (**13**)

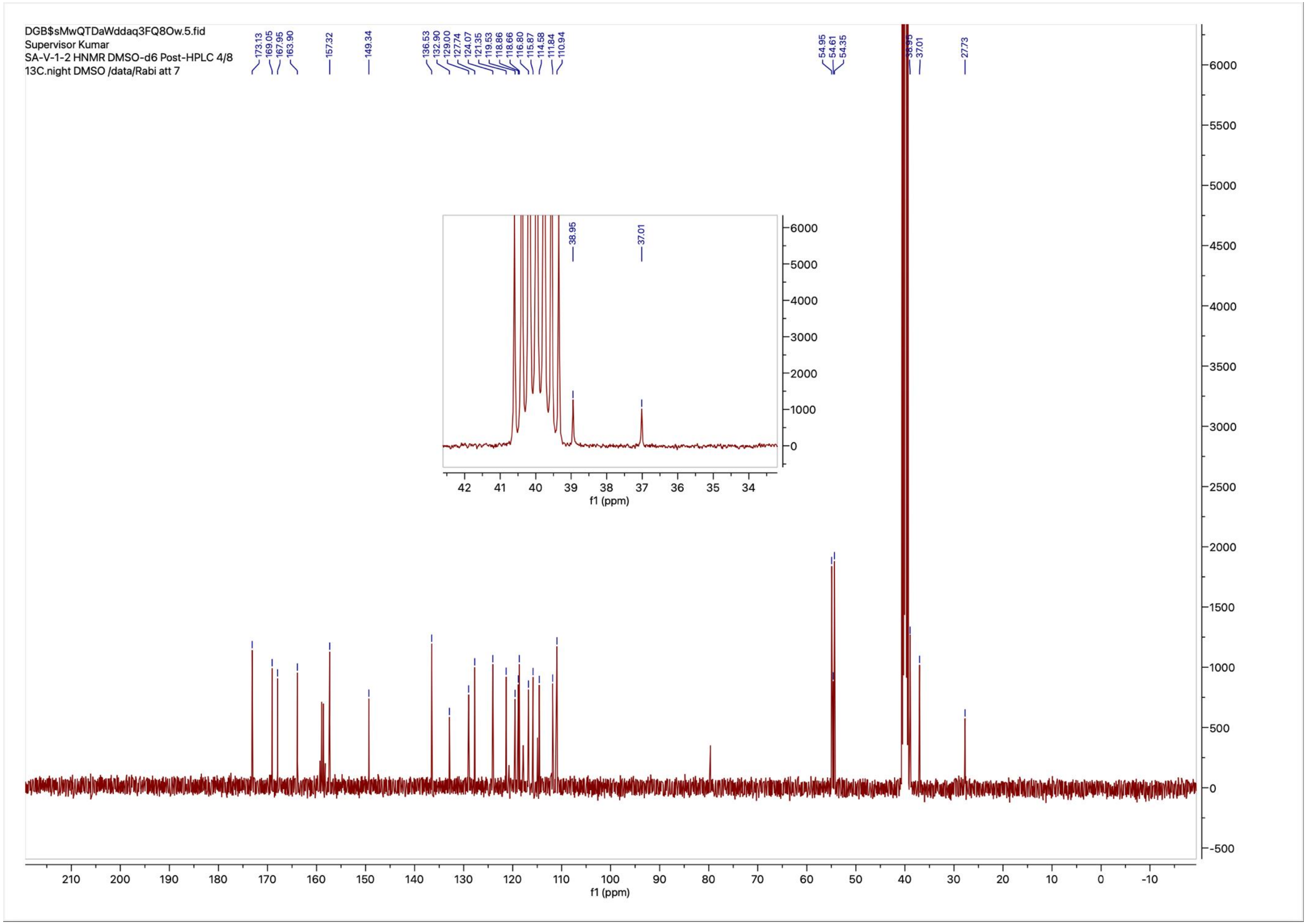

^{1}H NMR of (*S*)-3-((1-((2-ammonioethyl)amino)-3-(1*H*-indol-3-yl)-1-oxopropan-2-yl)carbamoyl)-[1,1':2',1''-terphenyl]-4-aminium (**14**)

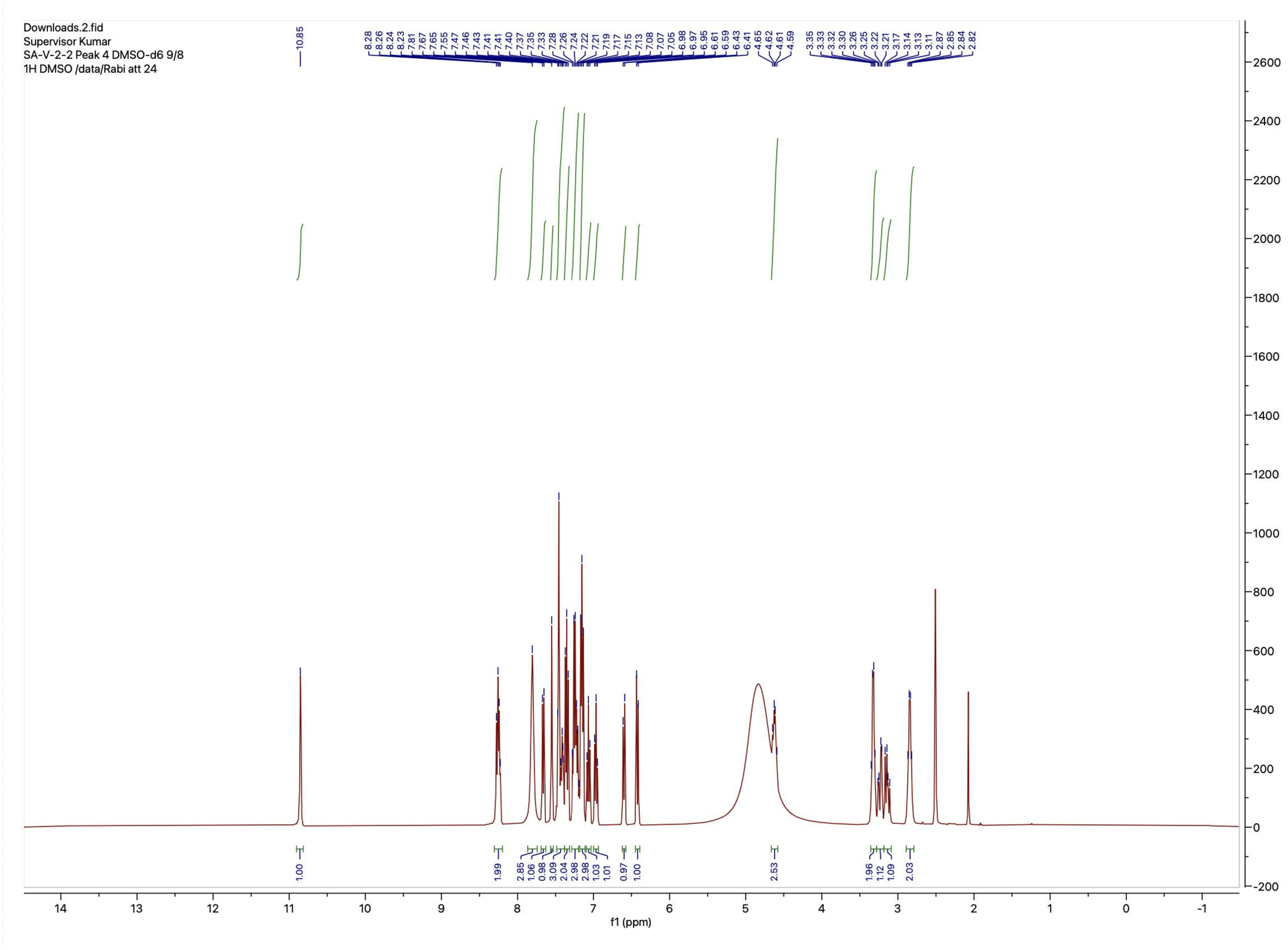

^{13}C NMR of (*S*)-3-((1-((2-ammonioethyl)amino)-3-(1*H*-indol-3-yl)-1-oxopropan-2-yl)carbamoyl)-[1,1':2',1''-terphenyl]-4-aminium (**14**)

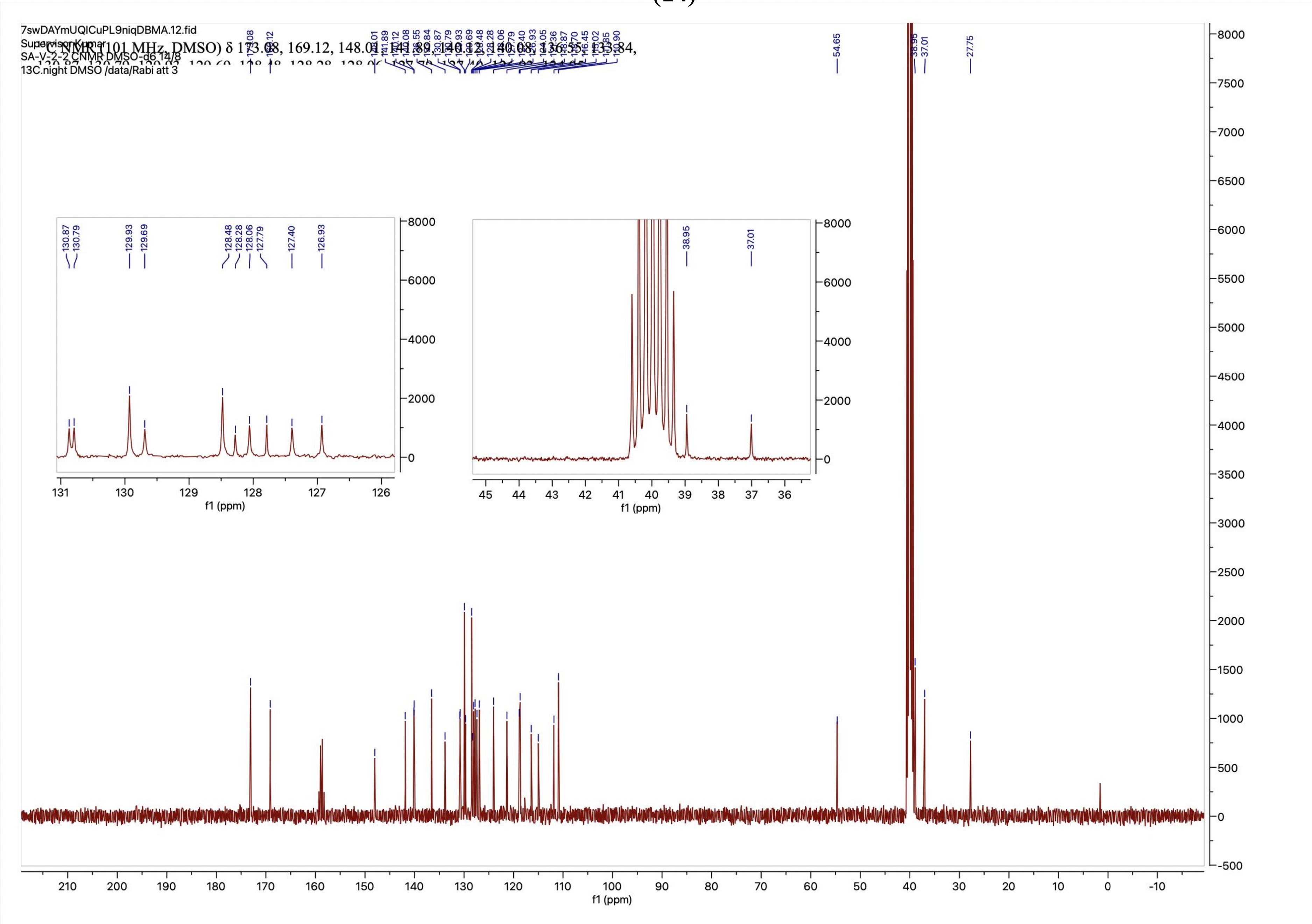

^{1}H NMR of *N*-(4-bromo-2-((((*S*)-1-((2-((*S*)-2,6-diaminohexanamido)ethyl)amino)-3-(1*H*-indol-3-yl)-1-oxopropan-2-yl)carbamoyl)phenyl)-2-naphthamide (**15**)

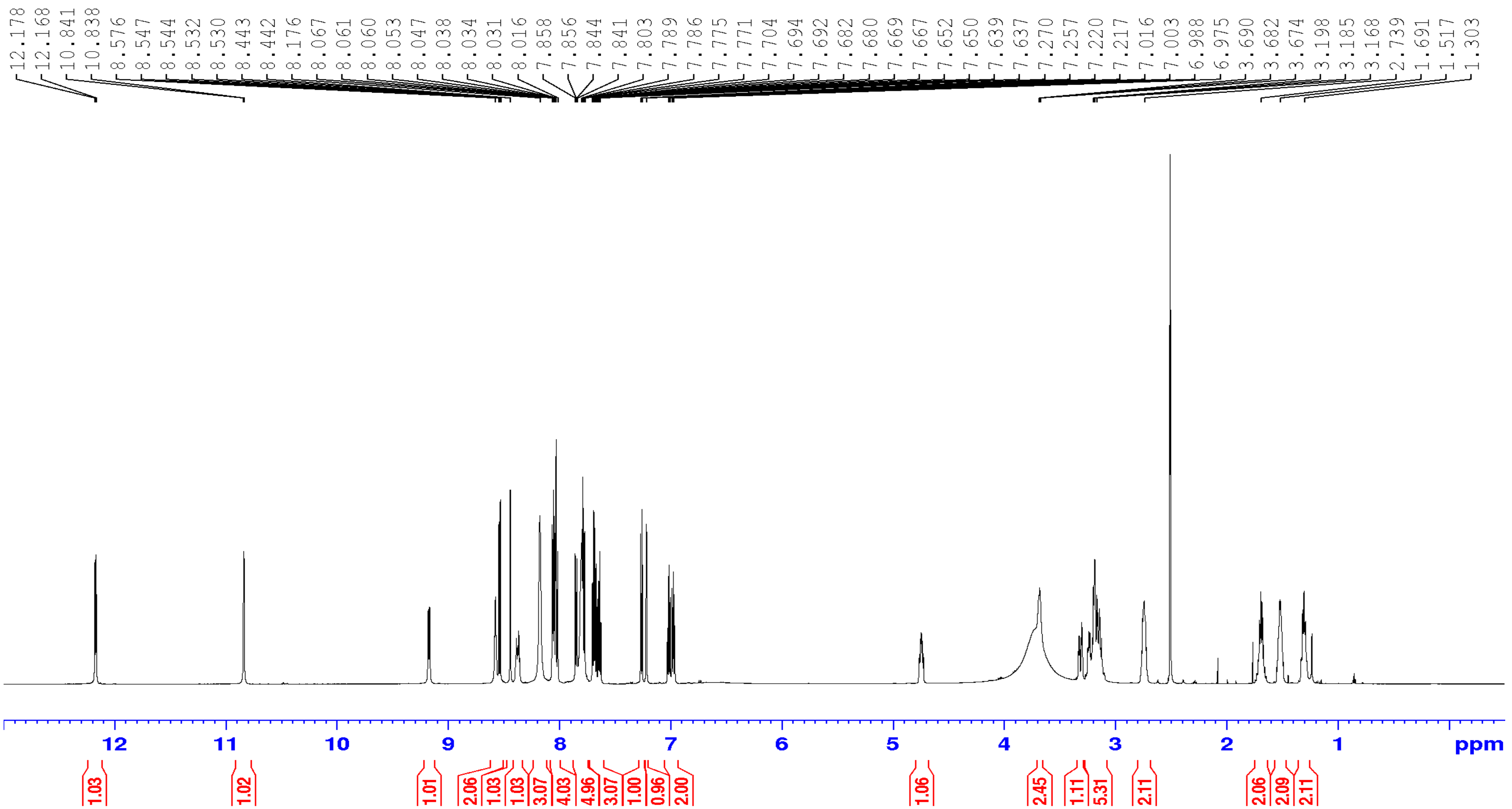

^{13}C NMR of *N*-(4-bromo-2-(((S)-1-((2-((S)-2,6-diaminohexanamido)ethyl)amino)-3-(1*H*-indol-3-yl)-1-oxopropan-2-yl)carbamoyl)phenyl)-2-naphthamide (**15**)

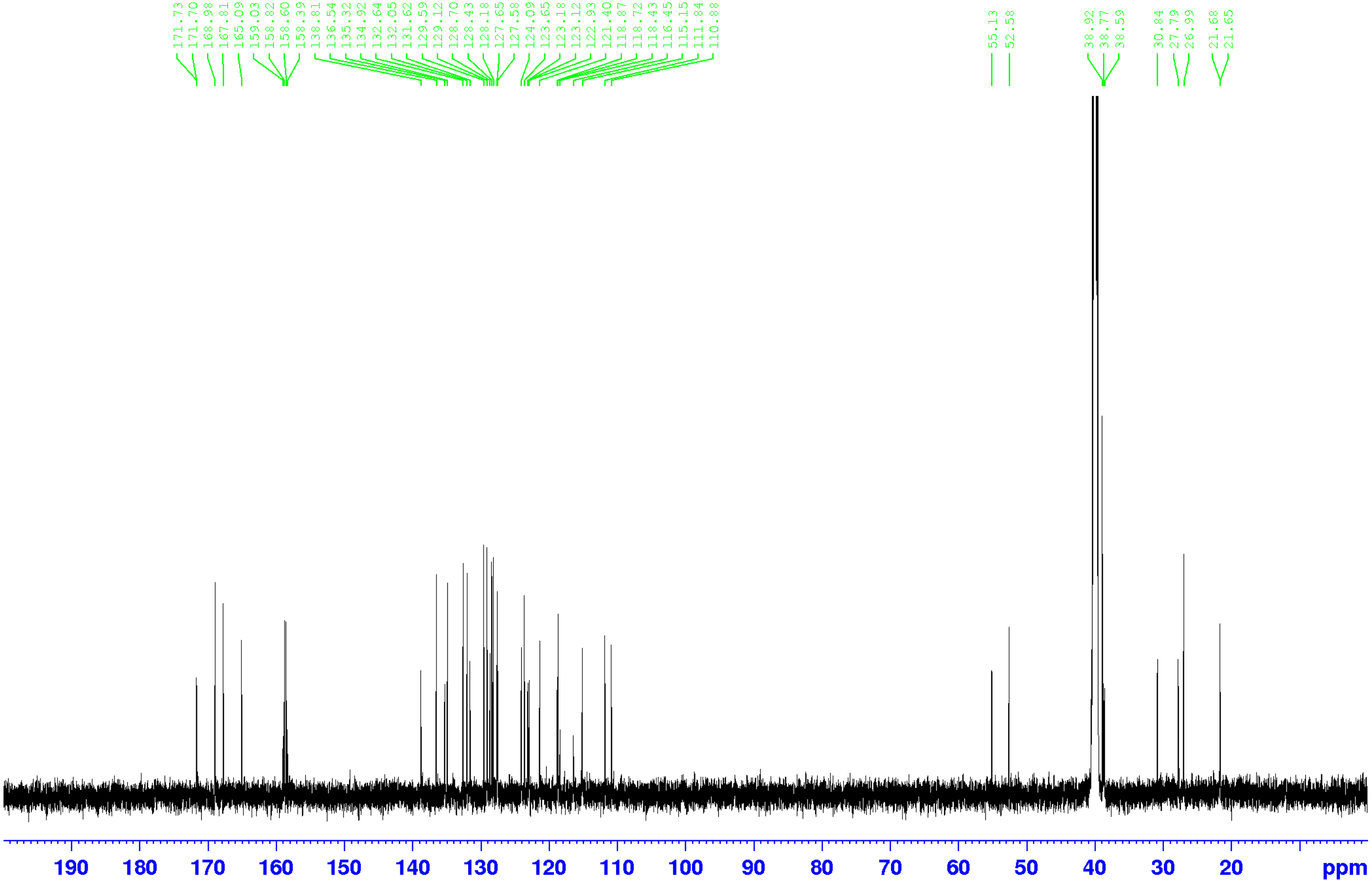

^{1}HNMR of *N*-(4-bromo-2-(((S)-1-((2-((S)-2,6-diaminohexanamido)ethyl)amino)-3-(1*H*-indol-3-yl)-1-oxopropan-2-yl)carbamoyl)phenyl)-[1,1'-biphenyl]-3-carboxamide (**16**)

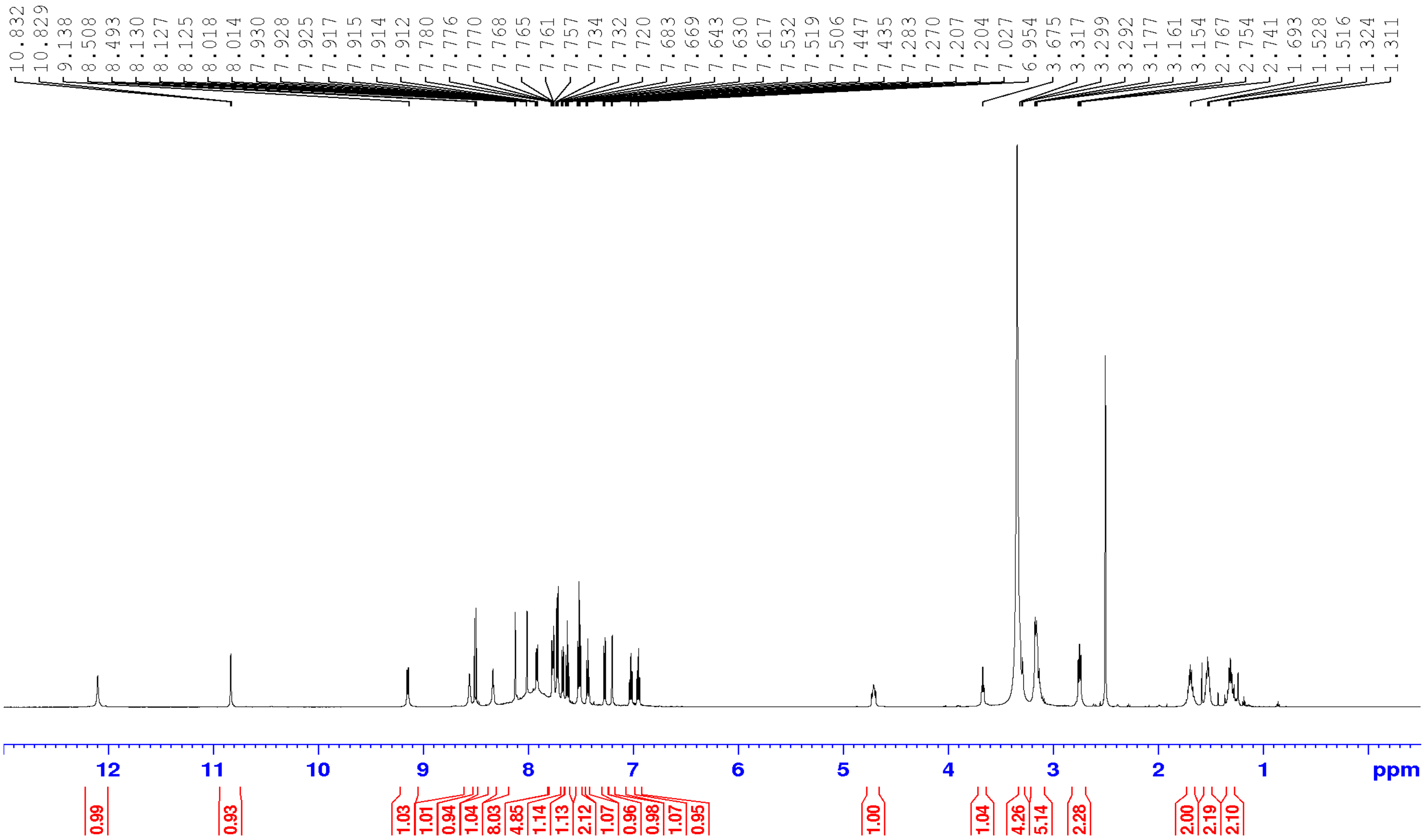

^{13}C NMR of *N*-(4-bromo-2-(((S)-1-((2-((S)-2,6-diaminohexanamido)ethyl)amino)-3-(1*H*-indol-3-yl)-1-oxopropan-2-yl)carbamoyl)phenyl)-[1,1'-biphenyl]-3-carboxamide (**16**)

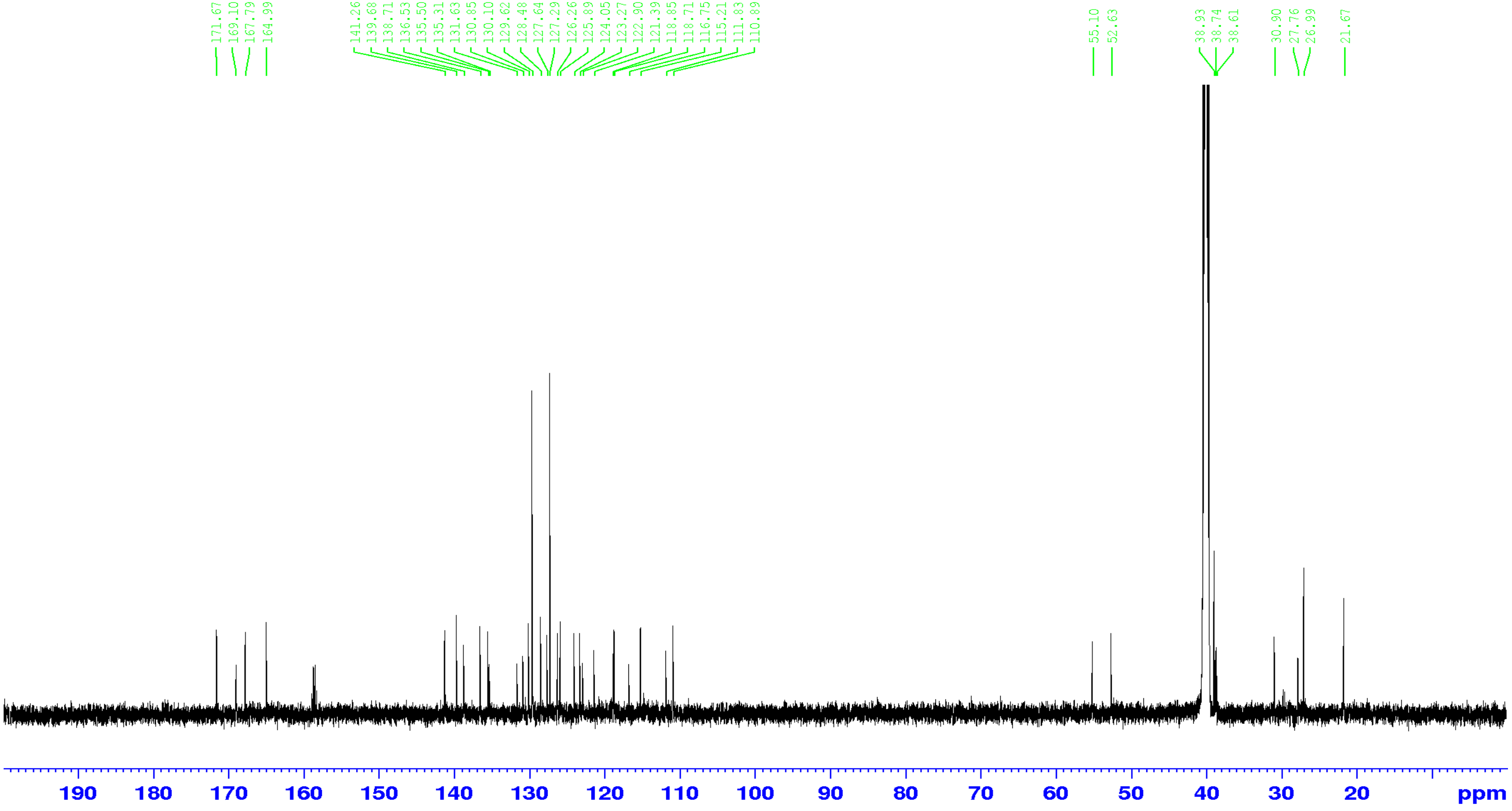

^{1}H NMR of *N*-(4-bromo-2-(((S)-1-((2-((S)-2,6-diaminohexanamido)ethyl)amino)-3-(1*H*-indol-3-yl)-1-oxopropan-2-yl)carbamoyl)phenyl)-[1,1'-biphenyl]-4-carboxamide (**17**)

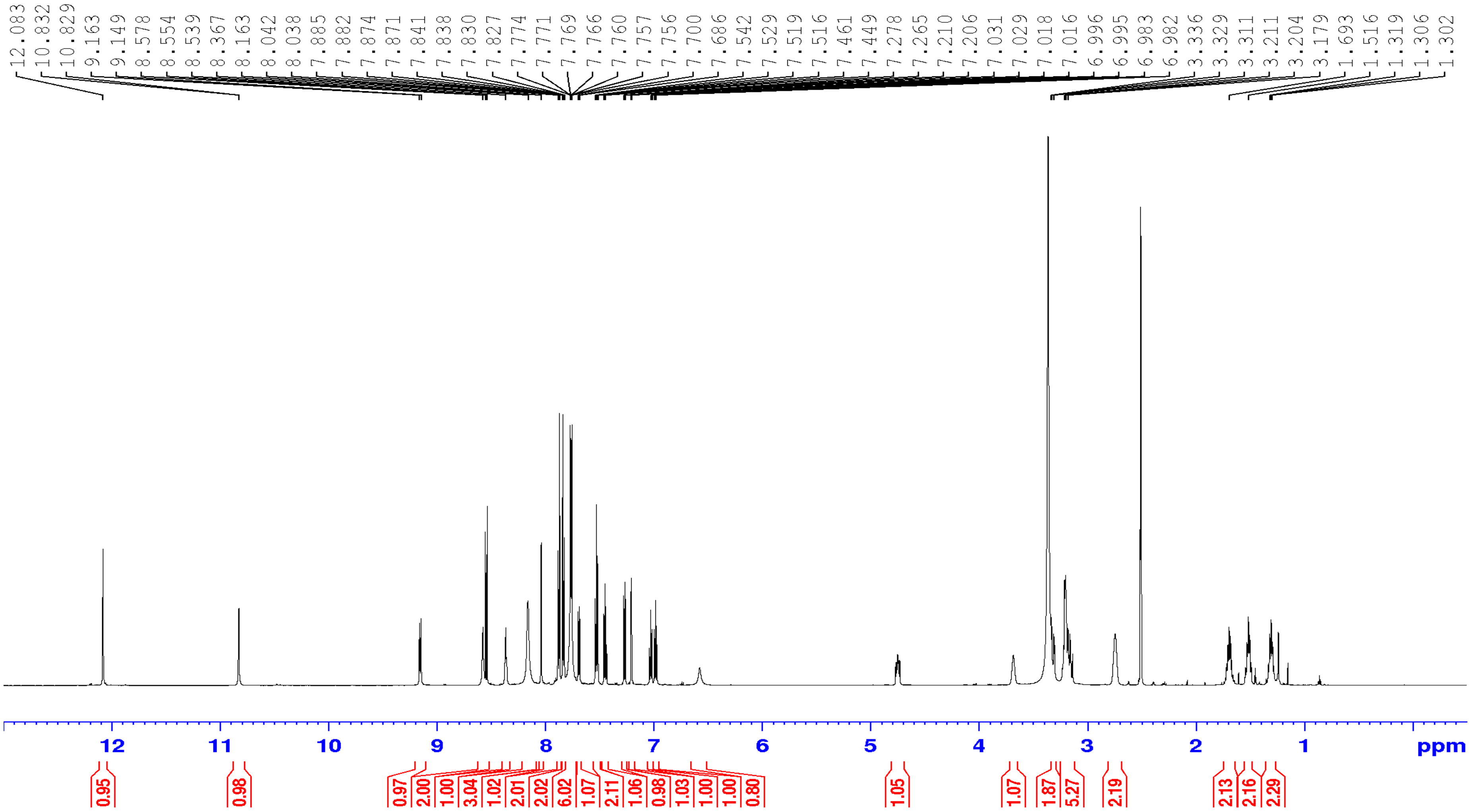

^{13}C NMR of *N*-(4-bromo-2-(((S)-1-((2-((S)-2,6-diaminohexanamido)ethyl)amino)-3-(1*H*-indol-3-yl)-1-oxopropan-2-yl)carbamoyl)phenyl)-[1,1'-biphenyl]-4-carboxamide (**17**)

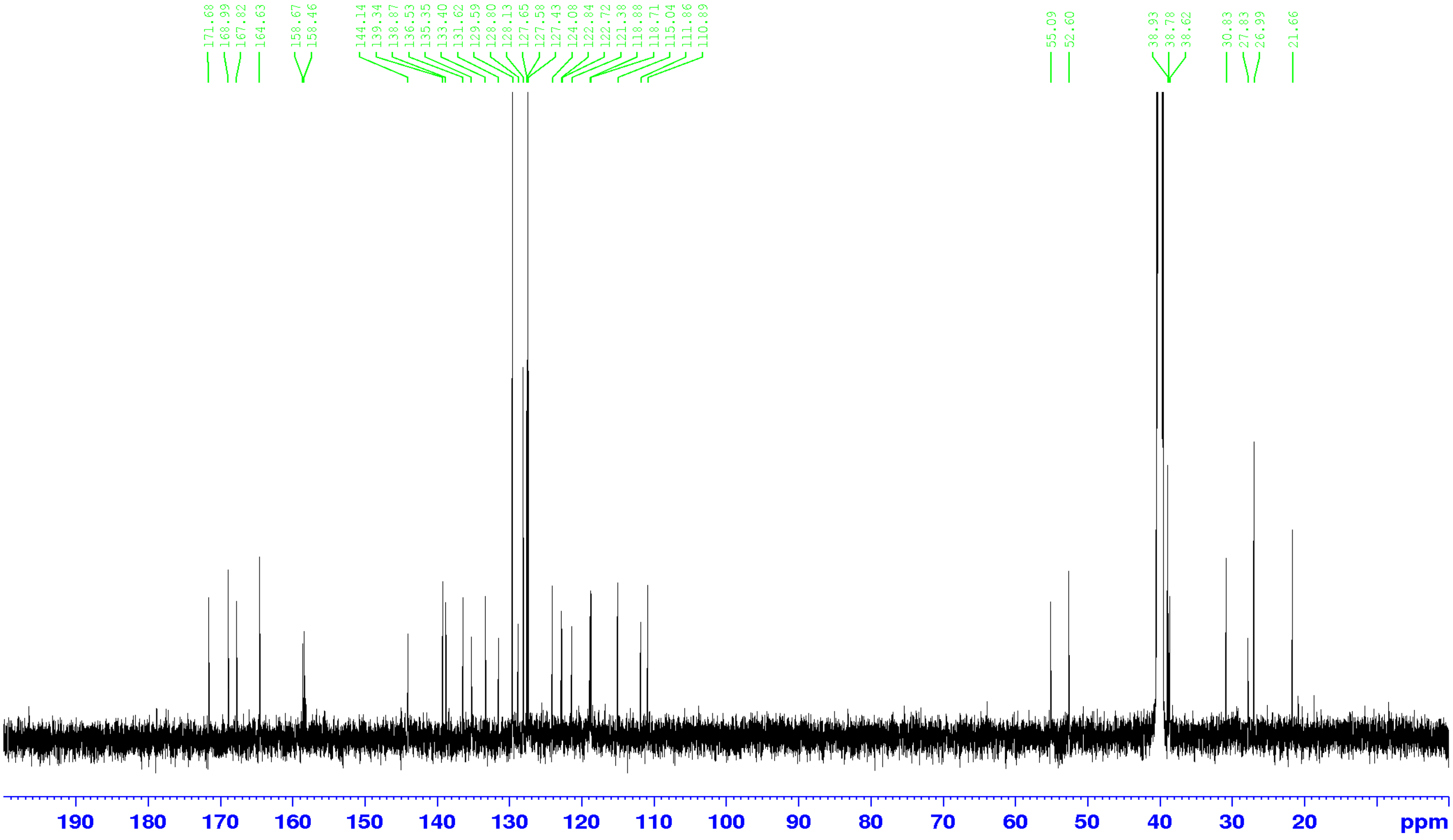

Supplement: Supplementary file 1 [file antibiotics-12-01436-s001.zip › antibiotics-2605419-supplementary.pdf]
